# The Multilayer Connectome of *Caenorhabditis elegans*

**DOI:** 10.1371/journal.pcbi.1005283

**Published:** 2016-12-16

**Authors:** Barry Bentley, Robyn Branicky, Christopher L. Barnes, Yee Lian Chew, Eviatar Yemini, Edward T. Bullmore, Petra E. Vértes, William R. Schafer

**Affiliations:** 1 Neurobiology Division, MRC Laboratory of Molecular Biology, Cambridge, United Kingdom; 2 HHMI Janelia Research Campus, Ashburn, VA, United States of America; 3 Department of Biological Sciences, Columbia University, New York, NY, United States of America; 4 Department of Psychiatry, University of Cambridge, Cambridge United Kingdom; 5 ImmunoPsychiatry, Alternative Discovery & Development, GlaxoSmithKline R&D, Cambridge United Kingdom; Oxford University, UNITED KINGDOM

## Abstract

Connectomics has focused primarily on the mapping of synaptic links in the brain; yet it is well established that extrasynaptic volume transmission, especially via monoamines and neuropeptides, is also critical to brain function and occurs primarily outside the synaptic connectome. We have mapped the putative monoamine connections, as well as a subset of neuropeptide connections, in *C*. *elegans* based on new and published gene expression data. The monoamine and neuropeptide networks exhibit distinct topological properties, with the monoamine network displaying a highly disassortative star-like structure with a rich-club of interconnected broadcasting hubs, and the neuropeptide network showing a more recurrent, highly clustered topology. Despite the low degree of overlap between the extrasynaptic (or wireless) and synaptic (or wired) connectomes, we find highly significant multilink motifs of interaction, pinpointing locations in the network where aminergic and neuropeptide signalling modulate synaptic activity. Thus, the *C. elegans* connectome can be mapped as a multiplex network with synaptic, gap junction, and neuromodulator layers representing alternative modes of interaction between neurons. This provides a new topological plan for understanding how aminergic and peptidergic modulation of behaviour is achieved by specific motifs and loci of integration between hard-wired synaptic or junctional circuits and extrasynaptic signals wirelessly broadcast from a small number of modulatory neurons.

## Author Summary

Connectomics represents an effort to map brain structure at the level of individual neurons and their synaptic connections. However, neural circuits also depend on other types of signalling between neurons, such as extrasynaptic modulation by monoamines and peptides. Here we present a draft monoamine connectome, along with a partial neuropeptide connectome, for the nematode *C*. *elegans*, based on new and published expression data for biosynthetic genes and receptors. We describe the structural properties of these "wireless" networks, including their topological features and modes of interaction with the wired synaptic and gap junction connectomes. This multilayer connectome of *C*. *elegans* can serve as a prototype for understanding the multiplex networks comprising larger nervous systems, including the human brain.

## Introduction

The new field of connectomics seeks to understand the brain by comprehensively mapping the anatomical and functional links between all its constituent neurons or larger scale brain regions [1]. The *C*. *elegans* nervous system has served as a prototype for analytical studies of connectome networks, since the synaptic connections made by each of its 302 neurons have been completely mapped at the level of electron microscopy [2, 3]. Through this approach, the *C*. *elegans* nervous system has been found to share a number of topological features in common with most other real-world networks, from human brain networks through social networks to the internet [1, 4, 5]. One well-known example is the small-world phenomenon, whereby networks are simultaneously highly clustered (nodes that are connected to each other are also likely to have many nearest neighbours in common) and highly efficient (the average path length between a pair of nodes is short) [6, 7]. Another characteristic feature of real-world networks which has attracted much attention is the existence of hubs or high-degree nodes, with many more connections to the rest of the network than expected in a random graph [8]. As in other networks, these topological features of the *C*. *elegans* connectome are thought to reflect the functional needs of the system [9, 10]. For example hubs are known to play a privileged role in coordinating functions across a distributed network [11], while the short path lengths (often mediated by the hubs) help increase the efficiency of information transfer across the network [6].

Although connectomics has primarily focused on mapping the synaptic links between neurons, it is well established that chemical synapses are only one of several modes of interaction between neurons. For example, gap junctions, which mediate fast, potentially bidirectional electrical coupling between cells, are widespread in all nervous systems. Likewise, volume transmission and neurohumoral signalling provide means for local or long-range communication between neurons unconnected by synapses. As neuromodulators released through these routes can have profound effects on neural activity and behaviour [12–14], a full understanding of neural connectivity requires a detailed mapping of these extrasynaptic pathways.

In *C*. *elegans*, as in many animals, one important route of neuromodulation is through monoamine signalling. Monoamines are widespread throughout phyla, with evidence that they are one of the oldest signalling systems, evolving at least 1 billion years ago [15]. In both humans and *C*. *elegans*, many neurons expressing aminergic receptors are not post-synaptic to releasing neurons, indicating that a significant amount of monoamine signalling occurs outside the wired connectome [16]. Monoamines are known to be essential for normal brain function, with abnormal signalling being implicated in numerous neurological and psychiatric conditions [17]. In *C*. *elegans*, these monoaminergic systems play similarly diverse roles in regulating locomotion, reproduction, feeding states, sensory adaptation, and learning [16]. Clearly, if the goal of connectomics is to understand behaviourally relevant communication within the brain, extrasynaptic monoamine interactions must also be mapped, not just the network of wired chemical synapses and gap junctions.

In addition to monoamines, neuropeptides are also widely used as neuromodulators in the *C*. *elegans* nervous system. *C*. *elegans* contains over 250 known or predicted neuropeptides synthesized from at least 122 precursor genes, and over 100 putative peptide receptors [18, 19]. These include homologues of several well-known vertebrate neuropeptide receptors, including those for oxytocin/vasopressin (NTR-1), neuropeptide Y (NPR-1) and cholecystokinin (CKR-2) [19]. As in other animals, neuropeptide signalling is critical for nervous system function, and frequently involves hormonal or other extrasynaptic mechanisms.

This study describes a draft connectome of extrasynaptic monoamine signalling in *C*. *elegans*, as well as a partial network of neuropeptide signalling, based on new and published gene expression data. We find that the extrasynaptic connectomes exhibit topological properties distinct from one another as well as from the wired connectome. Overall, the neuronal connectome can be modelled as a multiplex network with structurally distinct synaptic, gap junction, and extrasynaptic (neuromodulatory) layers representing neuronal interactions with different dynamics and polarity, and with critical interaction points allowing communication between layers. This network represents a prototype for understanding how neuromodulators interact with wired circuitry in larger nervous systems and for understanding the organisational principles of multiplex networks.

## Results

### A network of extrasynaptic monoamine signalling

To investigate the extent of extrasynaptic signalling in *C*. *elegans* monoamine systems, we systematically compared the expression patterns of monoamine receptors with the postsynaptic targets of aminergic neurons. Monoamine-producing cells were identified based on the published expression patterns of appropriate biosynthetic enzymes and vesicular transporters (see Methods). The expression patterns for each of five serotonin receptors (*ser-1*, *ser-4*, *ser-5*, *ser-7* and *mod-1*), three octopamine receptors (*octr-1*, *ser-3* and *ser-6*), four tyramine receptors (*ser-2*, *tyra-2*, *tyra-3* and *lgc-55*), and four dopamine receptors (*dop-1*, *dop-2*, *dop-3* and *dop-4*) were compiled from published data (see S1–S7 Tables). Since these receptors are either ion channels or serpentine receptors predicted to couple to pan-neuronal G-proteins, we therefore assumed all neurons expressing monoamine receptors are potential monoamine-responding cells.

**Fig 1:**
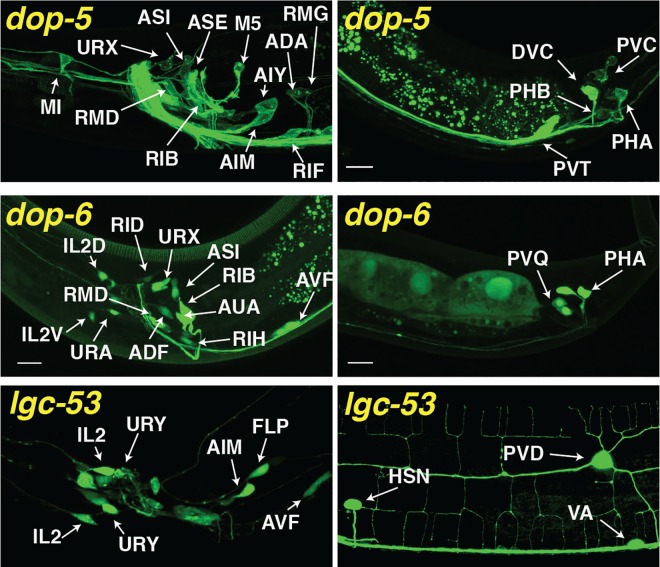
Expression patterns of the dopamine receptors *dop-5*, *dop-6* & *lgc-53*. Shown are representative images showing expression of GFP reporters under the control of indicated receptor promoters in the head (left panels) or tail/posterior body (right panels). Identified neurons are labelled; procedures for confirmation of cell identities are described in methods. In all panels, dorsal is up and anterior is to the left. In addition to the neurons indicated, dopamine receptor reporters were detected in the following neurons: *dop-5*: BDU (some animals); *lgc-53*: CAN (some animals).

Three additional genes encode known or candidate monoamine receptors but have missing or incomplete expression data. Specifically, a ligand-gated chloride channel, *lgc-53*, has been shown to be activated by dopamine [20], but its expression pattern and biological function have not been characterized. Additional expression profiling using a transgenic *lgc-53* reporter line crossed to a series of known reference strains indicated that *lgc-53* is expressed in a small subset of neurons in the head, body and tail (Fig 1). Together with the published *dop-1*, *dop-2*, *dop-3* and *dop-4*-expressing cells, these were inferred to make up the domain of dopamine-responding neurons. In addition, two G-protein coupled receptors, *dop-5* and *dop-6*, have been hypothesized based on sequence similarity to *dop-3* to be dopamine receptors. Using the same approach used for *lgc-53*, we identified most of the cells with clear expression of *dop-5* and *dop-6* reporters (Fig 1). These cells were included in a broader provisional dopamine network, the analysis of which is presented in the supplemental material (S1 Fig, S3 Fig).

**Fig 2:**
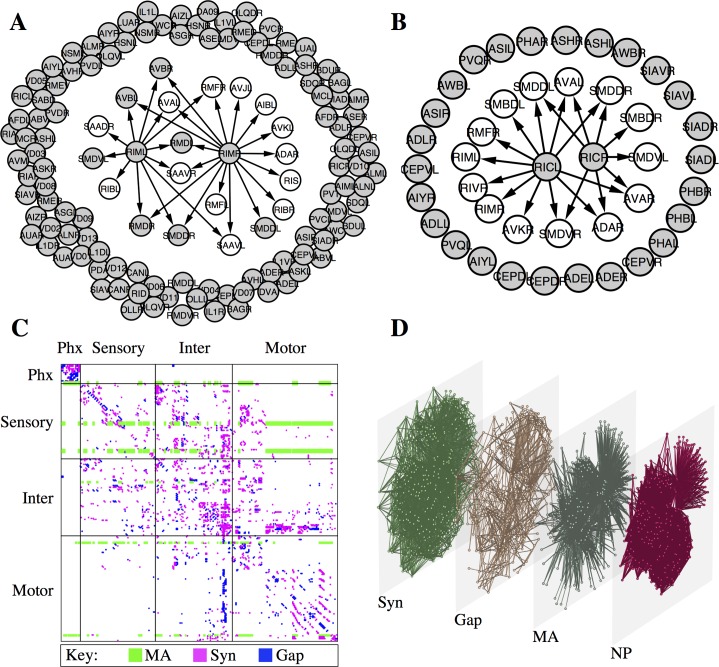
Monoamine signalling in *C*. *elegans* is primarily extrasynaptic. (A) RIM tyramine releasing neurons, showing outgoing synaptic edges (arrows), and neurons expressing one or more of the four tyramine receptors (grey). (B) RIC octopamine releasing neurons, showing outgoing synaptic edges (arrows), and neurons expressing one or more of the three octopamine receptors (grey). (C) Adjacency matrix showing the monoamine (green), synaptic (magenta) and gap junction (blue) networks. (D) Multilayer expansion of the synaptic (syn), gap junction (gap), monoamine (MA) and neuropeptide (NP) signalling networks. Node positions are the same in all layers.

Receptor expression patterns suggest that a remarkably high fraction of monoamine signalling must be extrasynaptic. For example, the two tyraminergic neurons, RIML and RIMR, are presynaptic to a total of 20 neurons. Yet of the 114 neurons that express reporters for one or more of the four tyramine (TA) receptors, only 7 are postsynaptic to a tyraminergic neuron (Fig 2A; Table 1). Thus, approximately 94% of tyramine-responsive neurons must respond only to extrasynaptic TA. Similar analyses of the other monoamine systems yield comparable results: 100% of neurons expressing octopamine receptors receive no synaptic input from octopamine-releasing neurons (Fig 2B), while 82% of neurons expressing dopamine receptors, and 76% of neurons expressing serotonin receptors receive no synaptic input from neurons expressing the cognate monoamine ligand (Table 1). Thus, most neuronal monoamine signalling in *C*. *elegans* appears to occur extrasynaptically, outside the wired synaptic connectome. The prevalence of extrasynaptic monoamine signalling between neurons unconnected by synapses or gap junctions implies the existence of a large *wireless* component to the functional *C*. *elegans* connectome, the properties of which have not previously been studied.

**Table 1:** Table showing the number of monoamine receptor-expressing cells that do not receive synapses from releasing cells, and the number of connections in each layer that are non-synaptic, including connections between neurons within the same class. Due to a many-to-many relationship between senders and receivers, the fraction of non-synaptic edges can exceed the fraction of non-synaptic cells/nodes. Values for the expanded network including putative *dop-5* and *dop-6* connections are in parentheses.

Network	Cells with no synaptic input		Non-synaptic edges	
	№	%	№	%
Serotonin	62	75.6	457	93.3
Dopamine	121 (138)	82.3 (73.8)	1133 (1422)	97 (95.6)
Octopamine	28	100	54	100
Tyramine	107	93.9	216	94.7
*Aggregate*	183 (178)	77.9 (70.9)	1860 (2149)	95.9 (95.1)

Using the gene expression data, a directed graph representing a draft aminergic connectome was constructed with edges linking putative monoamine releasing cells (expressing monoamines, biosynthetic enzymes, or transporters) to those cells expressing a paired receptor (Fig 2C; Table 2; S1 Dataset). Since biologically-relevant long-distance signalling (e.g. from releasing cells in the head to tail motoneurons) has been experimentally demonstrated in *C*. *elegans* for both dopamine and serotonin [21, 22]–while tyramine and octopamine are each released from a single neuronal class [16]–edges were not restricted based on the physical distance between nodes. For the serotonin network, only those neurons with strong, consistent expression of serotonin biosynthetic markers such as tryptophan hydroxylase were included (NSM, HSN and ADF). Additional neurons (AIM, RIH, VC4/5) that appear to take up serotonin but not synthesize it [23][24] were not included in the network, since they may function primarily in the homeostatic clearing of serotonin. We also did not include the ASG neurons, which produce serotonin only under hypoxic conditions [25], though they are likely to participate conditionally in the serotonin signalling networks.

**Table 2:** Table showing the number of nodes and edges in the individual and aggregate monoamine networks. Values for an expanded network including putative *dop-5* and *dop-6* connections are in parentheses.

Network	Nodes *N*	№ ligand expressing	№ receptor expressing	Edges *M*^*→*^
Serotonin	86	6	82	490
Dopamine	147 (187)	8	147 (187)	1168 (1488)
Octopamine	28	2	28	54
Tyramine	116	2	114	228
*Aggregate*	237 (251)	18	235 (251)	1940 (2260)

### The *C*. *elegans* connectome forms a multiplex network with nonredundant layers

With the inclusion of the monoamine systems, the full *C*. *elegans* connectome can be considered as a multiplex or multilayer network [26], with each node representing a neuron and each layer of connections–synaptic, gap junction, and monoamine–characterized by distinct edge properties (Fig 2D). For example, chemical synapses represent unidirectional, wired connections that signal on a fast (ms) time scale, while gap junctions generate reciprocal electrical connections that function on an even faster time scale. In contrast, monoamine connections are wireless (with a single sending cell broadcasting to multiple receivers), slow (acting on a time scale of seconds or longer) and unidirectional [22, 27]. Conceptually, additional modes of signalling between neurons, such as peptide neuromodulation, could represent additional layers.

**Fig 3:**
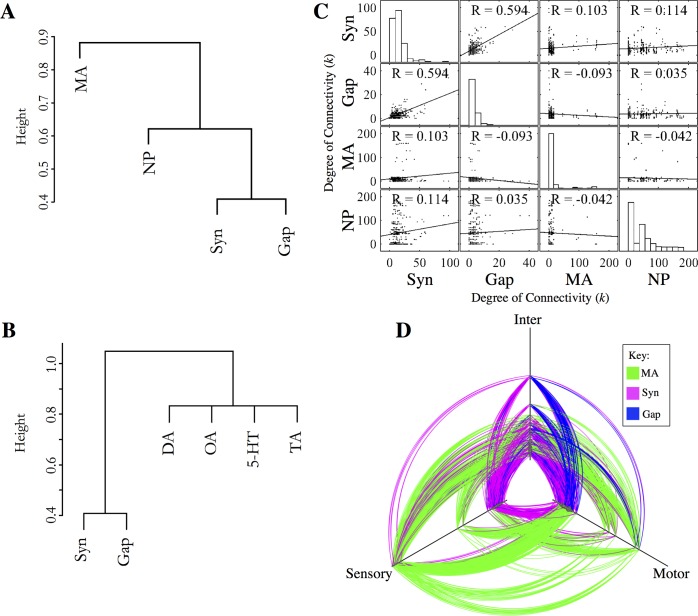
Monoamine networks are largely non-overlapping with the wired connectome. (A-B) Multilayer reducibility dendrograms. Panel A considers monoamine and neuropeptide networks in aggregate; panel B considers monoamine systems individually with neuropeptide systems not included. Layers close on the dendrogram have more overlapping edges and are more reducible. Branching height gives the Jensen-Shannon distance between the layers. (C) Degree-degree correlation matrix. Off-diagonal panels show the degree-degree correlation between a pair of network layers. Panels on the diagonal show the degree distribution of the individual layers. Monoamine hubs correspond to releasing neurons, which are distinct for each monoamine. (D) Hive plot showing the wired synaptic (magenta), gap junction (blue), and monoamine connections (green). Nodes are classified as sensory, motor or interneurons and are arranged along the three axes according to their degree. Hubs are located further out along the axes.

Prior studies of multiplex networks in non-biological systems–such as communication networks–have tended to find a large degree of overlap between the links observed in distinct layers, implying that they may not be truly independent channels of interaction [28]. In contrast, we observe that out of 1940 monoamine connections only 80 overlap with chemical or electrical synapses, meaning 96% of the monoamine connections are unique to the monoamine layer (Fig 2C; Table 1). Reducibility analysis [28], which clusters the different network layers based on their redundancy or degree of overlap, provides further support that the monoamine networks have a unique structure. Considered either separately or in the aggregate, the monoamines form a distinct cluster separate from the wired synaptic and gap junction networks (Fig 3A and 3B). This shows that the monoamine networks overlap less with the synaptic and gap junction networks than the synaptic and gap junction networks do with each other.

Similarly, in many previously-described multiplex networks, the high-degree hubs in each layer are often co-located, unequivocally highlighting certain nodes as key controllers of information flow in the system [26]. While the synaptic and gap junction layers of the worm connectome are observed to follow this trend, with the same high-degree neurons in both systems (Fig 3C), the extrasynaptic monoamine network exhibits a vastly different structure. While the synaptic and gap junction degrees of individual nodes show high positive correlation (R = .594), no significant degree-degree correlation is observed between the wired and extrasynaptic monoamine layers, indicating that the hubs of the monoamine system are distinct. These analyses suggest two distinct interpretations for the dissimilarity to the wired network layers. Firstly, monoamines may be functioning as an independent network, with little relation to the faster wired network. Secondly, the dissimilarity between layers might indicate that monoamines have a complementary function that is nevertheless coupled to that of the synaptic and gap junction connections.

### Analysis of monoamine network topology

**Fig 4:**
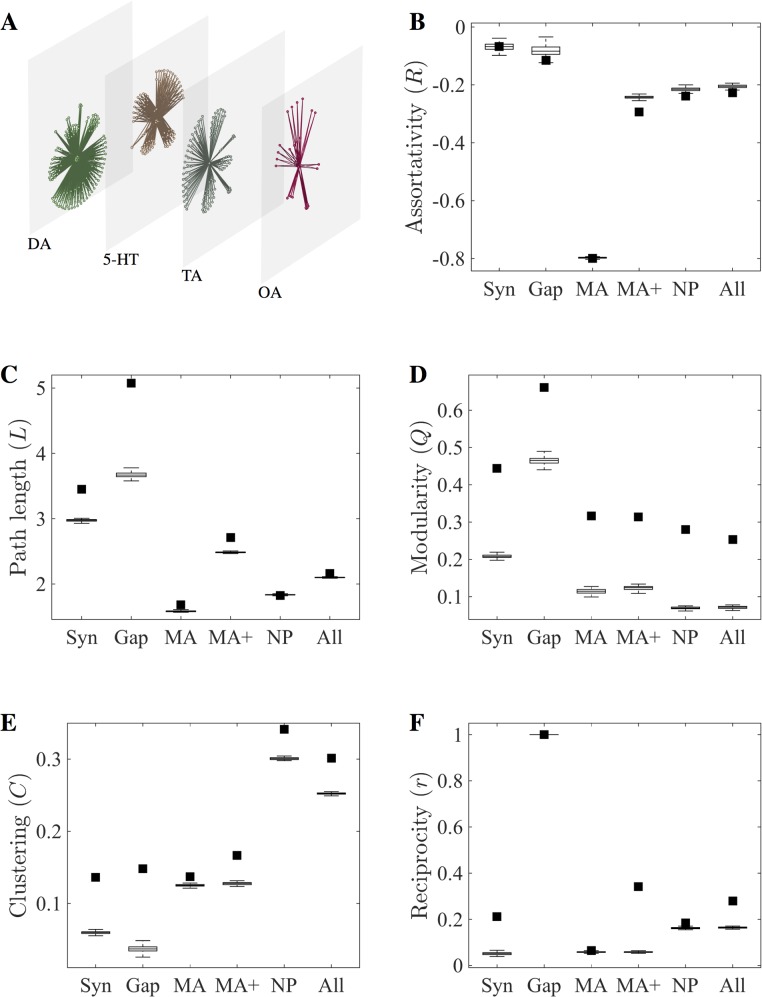
Topological properties of the *C*. *elegans* extrasynaptic networks. (A) Multilayer expansion of monoamine subnetworks. Node positions are the same in all layers. (B-F) Comparison of network metrics for the synaptic (syn), gap junction (gap), monoamine network (MA), aggregate wired & monoamine network (MA+), neuropeptide (NP) and complete aggregate (all) networks. Plots show the observed values (filled squares) and expected values for 100 rewired networks preserving degree distribution (boxplots). Network measures for individual monoamine networks and *dop-5/6*-containing aggregate network are presented in S1 Fig.

To address these possibilities, we investigated whether the isolated *C*. *elegans* monoamine network displays the structural organisation required for information processing. Considered separately, the monoamine networks of *C*. *elegans* consist of only a few topologically central neurons that broadcast signals to a large number of peripheral neurons. These monoamine-releasing cells are mostly sensory and motor neurons, with the downstream receptors being distributed throughout the worm (Fig 3D). In total, 18 of the 302 neurons in the adult hermaphrodite release monoamines, while 251 neurons (83%) were found to express one or more monoamine receptors. This gives the network a star-like topology, which can be directly observed in all of the separate monoamine networks (Fig 4A, S1 Fig). As a consequence, the monoamine network exhibits a heavy tailed distribution containing a small number of high-degree hubs (Fig 3C). This structure is also reflected in other topological network measures, with the monoamine network exhibiting high disassortativity characteristic of star networks (Fig 4B). Disassortativity is known to be relevant in the organisation of collective network dynamics, such as synchronisation [29] and cooperation behaviour [30, 31], and is widely observed in other biological and technological networks [32]. The star-like structure of the monoamine layer was also confirmed by three-neuron motif analysis, which revealed the enrichment of a motif consisting of a hub node signalling to two spokes (S2 Fig).

The inclusion of these additional monoamine connections into the connectome has a number of effects on the aggregate network. For one, it greatly reduces the overall path length of the network (Fig 4C), increasing the efficiency of integrative information processing by providing paths between more segregated subgraphs of the wired network [33]. In particular, monoamine signalling provides a direct route of communication between sensory neurons and motor neurons (Fig 3D), bypassing the premotor interneurons that play a prominent role in the synaptic and gap junction systems [11]. Together, these observations suggest that the monoamines provide efficient global connections for coordinating behaviour throughout the entire organism due to the presence of highly connected hubs directly linking many disparate parts of the network. This is a useful feature given the role of monoamines in signalling physiologically important states relevant to the entire organism, such as food availability [27]. The increased connectivity provided by the monoamines also results in a reduction in the aggregate network's modular structure, a consequence of increasing the number of connections between functionally segregated units (Fig 4D). The network is, however, still more modular than random, with the monoamine layer also exhibiting greater-than-random modularity compared to null models that rewire the network edges while preserving degree distribution (see Methods). This is expected given the monoamine layer's composition from separate signalling systems; indeed the individual monoamine networks considered on their own show very low modularity (S1 Fig).

Despite the hub-and-spoke structure of the extrasynaptic network, the monoamine layer exhibits a significant level of global clustering (measured here as *transitivity*) (Fig 4E). This observation is explained by two factors. Firstly, the expression of monoamine receptors by releasing neurons creates a central cluster of hub neurons in the network; secondly, as many neurons also express more than one monoamine receptor, triangles are formed in the network with a receiving neuron as one vertex, and two transmitting neurons as the others. Indeed, three-neuron motif analysis confirmed that this configuration is overrepresented in all the monoamine networks save tyramine (S2 Fig). This structure provides a method of dual lateral inhibition, where a releasing neuron can inhibit antagonistic signals from another hub neuron while simultaneously negating the downstream effects of those signals, a pattern previously observed in the OA/TA and 5-HT systems between RIC/RIM & NSM in the aminergic control of feeding behaviours [34]. Similar patterns also exist within individual monoamine layers; for example, the ventral cord motor neurons express both excitatory (*dop-1*) and inhibitory (*dop-3*) dopamine receptors [35], while the expression of an inhibitory receptor (*dop-2*) in dopamine-releasing neurons suggests that the hubs mutually suppress one another to regulate dopamine release.

**Fig 5:**
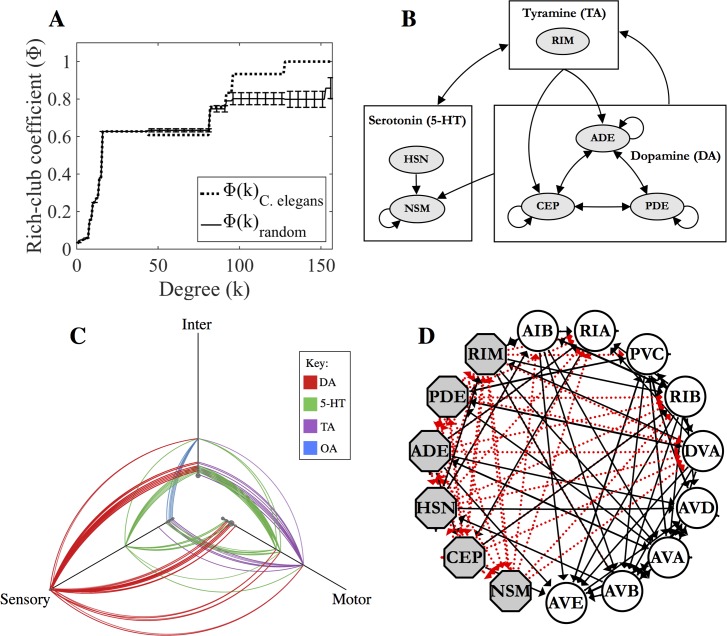
Monoamine rich-club. (A) Rich-club curve for the directed monoamine network. Dashed line indicates the rich-club coefficient for the *C*. *elegans* monoamine network and the solid curve is a randomized rich-club curve representing the average rich-club coefficient of 100 random graphs (preserving degree distribution) at each value *k*. Individual rich-club neurons are shown in Table 3. (B) Schematic showing the separate aminergic systems and the volume transmission signalling between them based on receptor expression. Arrows between boxes denote connections between all of the contained neurons. (C) Hive plot showing the connections made by individual monoamines. Nodes are classified as sensory, motor or interneurons and are arranged along the three axes according to their degree. Hubs are located further out along the axes. (D) Connections between the wired & monoamine rich-clubs. Aminergic rich-club neurons are represented as grey octagons. Members of the wired rich-club are shown as circles (RIBL but not RIBR is included due to its higher synaptic degree). Dashed red lines are extrasynaptic links. Solid black lines are chemical or electrical synapses.

Many neural and brain networks have been shown to exhibit rich-club organisation [36–39] in which the most highly-connected nodes are more connected to one another than expected by chance [40]. It was previously shown that the *C*. *elegans* wired connectome includes a rich-club consisting primarily of a small number of premotor interneurons, controlling forward and backward locomotion [11]. Subjecting the monoamine connectome to similar analysis, it was found that this network also contains a distinct rich-club (Fig 5A and 5B; Table 3), consisting of dopamine, serotonin, and tyramine-releasing neurons. The rich-club property stems from the fact that most serotonergic neurons contain receptors for both tyramine and dopamine, while dopaminergic and tyraminergic neurons likewise express receptors for the other two aminergic transmitters (Fig 5B), suggesting that the different monoamines coordinate their actions. This rich-club structure is also reflected in the 3-neuron motif analysis, in which the fully-connected motif was overrepresented in the aggregate monoamine layer (S2 Fig). Interestingly, in contrast to the wired rich-club, all of whose members are interneurons, the monoamine rich-club consists of sensory neurons and motor neurons (Fig 5C, Table 3).

**Table 3:** Rich-club neurons of the aggregate monoamine network. Number of neurons in each class are shown in parentheses next to the neuron ID. The rich-club column shows the threshold regime to which each neuron belongs, thus 3σ indicates Φ_norm_ (*k*) >1+3 σ, where σ is the standard deviation of the null model samples.

Neuron ID	Degree*k*_ma_	Rich-clubΦ_norm_	MA	Receptors	Type
CEP (4)	157	3σ	DA	*dop-2*, *octr-1*, *tyra-3*	Sensory
ADE (2)	157	3σ	DA	*dop-2*, *octr-1*, *tyra-3*	Sensory
PDE (2)	153	3σ	DA	*dop-2*	Sensory
RIM (2)	128	3σ	TA	*ser-4*, *mod-1*, *dop-1*	Motor
NSM (2)	96	3σ	5-HT	*ser-4*, *dop-3*, *ser-2*, *tyra-2*	Pharynx
HSN (2)	92	1σ	5-HT	*lgc-53*, *lgc-55*	Motor

### Properties of a partial neuropeptide network

**Fig 6:**
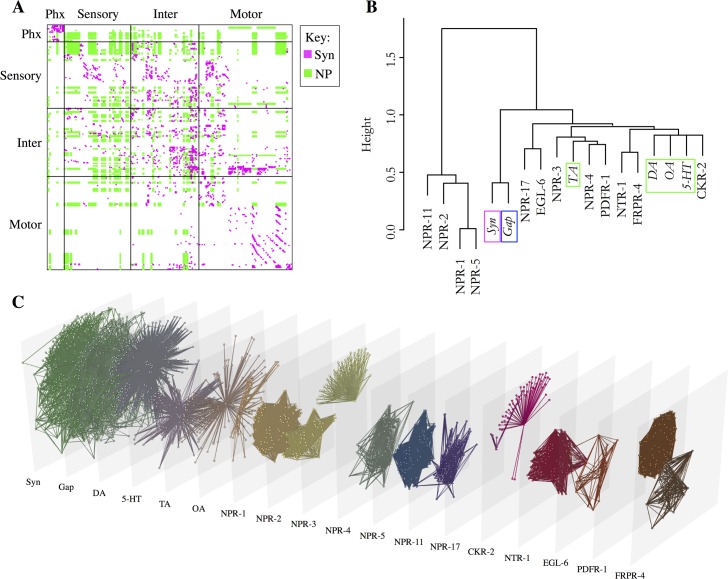
Neuropeptide networks. (A) Adjacency matrix showing the synaptic (magenta) and neuropeptide (green) networks. (B) Multilayer reducibility dendrograms for individual neuropeptide networks. Layers close on the dendrogram have more overlapping edges and are more reducible. Branching height gives the Jensen-Shannon distance between the layers. Wired and monoamine layers are italicized and indicated with green (MA), blue (gap junction), or magenta (synaptic) boxes. (C) Multilayer expansion of wired, monoamine, and neuropeptide networks. Node positions are the same in all layers.

We next investigated the structure of the signalling network for neuropeptides. The receptors for many neuropeptides, and the ligands for many neuropeptide receptors, remain unknown; moreover, the distance over which signalling can occur is uncharacterized for most neuropeptide systems. Despite these caveats, we reasoned that a partial and provisional neuropeptide network could provide useful insight into the differences between peptide signalling networks and synaptic, gap junction and monoamine networks. We focused on 12 neuropeptide receptors with well-established ligands (with biologically-plausible EC_50_ values in in vitro assays) and precisely-characterized expression patterns for both receptor and peptide precursor genes (S8 Table, S9 Table). Networks were classified by receptor, allowing many-to-many relationships between neuropeptides and receptors. Even for this partial network, 239 neurons are seen to be involved in neuropeptide signalling (out of 302 possible) with 7035 connections between them, providing greater connectivity than either the synaptic or monoamine layers. Of the receptor-expressing neurons, almost 60% received no synaptic input from neurons expressing one of their ligands, suggesting that at least for this partial network, neuropeptide signalling, like monoamine signalling, is largely extrasynaptic. Likewise, the majority of edges in the neuropeptide network do not overlap with synapses (97% non-overlapping), again consistent with a largely extrasynaptic mode of signalling (Fig 6A).

The neuropeptide network, like the monoamine network, exhibits a structure distinct from the wired connectome. No significant degree correlation was observed between the partial neuropeptide network and the synaptic, gap junction, or monoamine networks, indicating that neuropeptide hubs are distinct from those in other layers (Fig 3C). Likewise, reducibility analysis shows low overlap between the neuropeptide edges and those in the monoamine, synaptic and gap junction layers (Fig 3A). Interestingly, some individual neuropeptide systems, in particular CKR-2, overlap significantly with the networks of monoamine systems, while others, including the neuropeptide F/Y receptors NPR-1/2/5/11, show little overlap with either the wired or other extrasynaptic networks (Fig 6B).

Examining the network measures for the neuropeptide network reveal it to have some topological properties in common with the monoamine network, but also crucial differences. For example, both networks have a shorter characteristic path length and lower modularity than the wired networks (Fig 4C and 4D). On the other hand, the neuropeptide network has much higher clustering than any other connectome layer (Fig 4E), and is significantly less disassortative (Fig 4B) than the monoamine network. In part, this is an expected consequence of the large number of connections in the neuropeptide network; however, the observed clustering in the neuropeptide network was significantly higher even than null models with the same edge density. In addition, the neuropeptide network shows much higher reciprocity than the monoamine network (Fig 4F), with the individual neuropeptide systems generally lacking the star-like topology characteristic of the monoamines (Fig 6C).

### Modes of interaction between wired and extrasynaptic layers

Despite the distinct structures and topologies of the different neuronal connectome layers, they are likely to interact in functionally significant ways. For example, although the wired and monoamine rich-clubs do not overlap, there are significant links between them (Fig 5C). To systematically identify neurons that have a role in linking all of the layers, neurons were first ordered according to the product of their degree-rank across the synaptic, gap junction and monoamine layers (Table 4). We observe that the highest ranking neurons, which have the highest participation across all layers, include three from the monoamine rich-club (RIML, RIMR, and ADEL) and two from the wired rich-club (RIBL and DVA). Indeed, the premotor interneuron DVA is a receiver for serotonin, tyramine and (provisionally) dopamine signalling, while the tyraminergic RIMs are highly connected to the premotor interneurons of the wired rich-club. As one might expect from their topological role in linking the monoamine and wired network layers, the RIMs have been shown in a number of studies to play a central role in the modulation of sensory pathways in response to feeding states as well as the control of downstream locomotion motor programs [41–43]. Similarly RIB, which expresses receptors for serotonin and dopamine, is thought to integrate numerous sensory signals [44, 45] and has been demonstrated to influence reorientation in foraging behaviour [46].

**Table 4:** Multilayer hub neurons for 3-layer connectomes. The normalized degree product (*k*_norm_) showing the neurons with the highest degree rank across all of the layers. Rich-club neurons are indicated with ⋆

Neuron	*k*_norm_	*k*_syn_	*k*_gap_	*k*_ma_
RIMR⋆	0.236	34	14	128
RIBL⋆	0.207	29	30	14
RIBR	0.178	25	30	14
RIML⋆	0.171	28	12	128
RIS	0.119	27	16	14
ADEL⋆	0.073	31	4	157
VD01	0.070	14	16	16
DVA⋆	0.069	54	10	8
PVQR	0.069	22	10	16
AIBR	0.066	36	16	6

**Fig 7:**
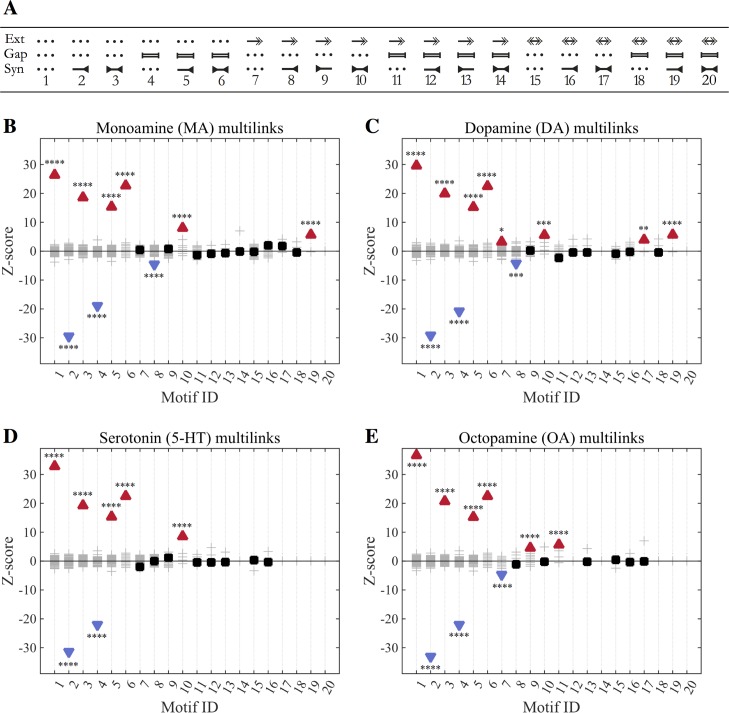
Modes of interaction between connectome layers. Shown are overrepresented and underrepresented multilink motifs for 3-layer networks consisting of synaptic, gap junction and monoamine (aggregate or individual MA) layers. (A) Multilink motif IDs. These correspond to all possible configurations of links between two neurons allowing for: no connection of a given type (dotted line), directed extrasynaptic monoamine links (Ext, represented as arrows on the top), bidirectional gap junctions (represented as bars in the middle) and synapses (represented as inverted arrowheads on the bottom line). (B-C) Motif *z*-scores for aggregate monoamines (B), dopamine (C), serotonin (D) or octopamine (E) 3-layer multilink. Plots for tyramine and *dop-5/6*-containing monoamine multilink are in S3 Fig. Over-represented motifs are represented by red upward-pointing triangles. Under-represented motifs are represented by blue downward-pointing triangles. Non-significant motifs are shown by black squares. Values for randomized null model networks are shown as grey crosses. Asterisks report the significance level using the z-test, with Bonferroni-adjusted p-values: * indicates *p* ≤ 0.05; ** indicates *p* ≤ 0.01; *** indicates *p* ≤ 0.001; **** indicates *p* ≤ 0.0001. Observed and expected multilink frequencies are in Table 5. Examples of monoamine *motif 10* are listed in Table 6.

Multilink motif analysis provides another approach for investigating the interactions between the synaptic, gap junction and monoamine layers [47]. Since each layer contains the same set of nodes but a different pattern of edges, the frequencies with which different combinations of links co-occur between pairs of nodes throughout the multiplex network can be determined. Of the 20 possible multilink motifs, seven were found to be overrepresented and four underrepresented compared to networks composed from randomized layers (Fig 7). Many of these do not involve monoamines; for example, three overrepresented motifs–reciprocal chemical synapses (*motif 3*) and the co-occurrence of a gap junction with a single or reciprocal chemical synapse (*motifs 5 & 6*)–have been reported in an earlier analysis of the wired network [5]. These also align with results from the degree-degree correlation and reducibility (Fig 3A, 3B and 3C) indicating that synapses and gap junctions frequently overlap. This is mirrored in the underrepresentation of *motifs 2 & 4* corresponding to synapses or gap junctions alone; conversely, the underrepresentation of these single link motifs leads to an overrepresentation of unlinked pairs (*motif 1*).

**Table 5:** Monoamine Multilinks. Multilink motif frequencies for the monoamine, synaptic and gap junction layers. Motif IDs correspond to those depicted in Fig 7.

Motif ID	Frequency	Expected	Z-score
1	41298	40974	26.43
2	1543	1991	-29.46
3	178	52	18.61
4	351	491	-18.95
5	154	54	15.39
6	49	4	22.68
7	1703	1698	0.48
8	52	78	-4.56
9	39	35	0.77
10	14	2	8.05
11	8	12	-1.29
12	0	1	-0.85
13	0	0	-0.63
14	0	0	-0.14
15	49	50	-0.25
16	11	6	2.04
17	1	0	1.83
18	0	0	-0.48
19	1	0	5.66
20	0	0	NaN

**Table 6:** Examples of monoamine multilink *motif 10*. List of neurons connected by *motif 10* (i.e. unidirectional MA link, no gap junctions, and reciprocal synapses). Examples involving unconfirmed (i.e. *dop-5* or *dop-6*-mediated) dopamine receptors are highlighted in grey.

Cell A		Cell B
NSM (L/R)	→	I6
ADFR	→	ASHR
ADFR	→	AWBR
ADEL	→	IL2L
ADE (L/R)	→	FLPL
ADER	→	FLPR
ADFR	→	AIYR
CEPDR	→	RIS
RIMR	→	RMDR
HSNL	→	AIAL
HSNR	→	AVJL
HSNR	→	PVQR
CEP (DL/VL)	→	OLLL
CEP (DR/VR)	→	OLLR
ADEL	→	BDUL
PDEL	→	DVA

Although the overlap between monoamine and wired connectivity is low, multilink motif analysis revealed a few overrepresented motifs involving monoamines. The most interesting (and statistically significant) of these corresponds to a unidirectional monoamine link coincident with reciprocal synaptic connections (*motif 10*). The structure of this motif is well-suited to provide positive or negative feedback in response to experience, suggesting that this may be a functionally important aspect of monoamine activity within the wider network. Indeed, connections of this type (Table 6) have been implicated in a number of *C*. *elegans* behaviours; for example, *motif 10* connections between ADF and AIY have been shown to be important for the learning of pathogen avoidance [48] and connections between RIM and RMD are important for the suppression of head movements during escape behaviour [49]. Putative *motif 10* connections between PDE and DVA are also thought to play a role in controlling neuropeptide release [50].

Intriguingly, most examples of *motif 10* (all except RIMR-RMDR) involve either serotonin or dopamine as the monoamine transmitter. Indeed, when we considered the monoamine networks separately (e.g. Syn-Gap-DA or Syn-Gap-TA multilink), *motif 10* was overrepresented for multilink containing either serotonin and dopamine (Fig 7C and 7D, S3 Fig), but not for tyramine or octopamine (Fig 7E, S3 Fig). Interestingly, two different motifs were found to be overrepresented in the 3-layer octopamine network (Fig 7E, S11 Table), *motif 9* (a unidirectional synaptic connection coincident with an octopamine connection in the opposite direction) and *motif 11* (a unidirectional octopamine link coincident with a gap junction). (Presumably these were not overrepresented in the aggregate network because the octopamine network is much smaller than the networks for the other monoamines). These motifs might serve similar functions to *motif 10* for dopamine and serotonin in providing feedback to modulate wired connections.

Interestingly, although the neuropeptide network showed little structural overlap with the monoamine network, its modes of interaction with the wired connectome showed striking parallels. When the neuropeptide network was included in the multiplex participation analysis, we observed that the RIM and DVA neurons continue to play central roles in linking the four network layers (S12 Table). Likewise, multilink motif analysis, this time using the neuropeptide and wired layers, again identified *motif 10* (a unidirectional neuromodulatory connection coincident with a reciprocal synaptic connection) as significantly overrepresented, further supporting the notion that this motif plays a key role in extrasynaptic modulation of synaptic computation (S3 Fig; S13 Table). Even more highly overrepresented relative to expectation was *motif 20*, reciprocal neuropeptide and synaptic connections coincident with a gap junction. This motif was not overrepresented in the multilink analysis for monoamines, perhaps because of the low reciprocity of the monoamine network. Interestingly, several of the *motif 20* multilink (S14 Table) are components of the RMG hub and spoke network, which has been implicated in the control of various behaviours including locomotion, aggregation, and pheromone response [51, 52].

## Discussion

This study has analysed the properties of an expanded *C*. *elegans* neuronal connectome, which incorporates newly-compiled networks of extrasynaptic monoamine and neuropeptide signalling. Analyses reveal that these extrasynaptic networks have structures distinct from the synaptic network, and from one another. The monoamine network has a highly disassortative, star-like topology, with a small number of high-degree broadcasting hubs interconnected to form a rich-club core. The monoamine systems are thus well-suited to broadly coordinate global neural and behavioural states across the connectome. Although the partial neuropeptide network we analyse is only a small sample of the complete network, it shows a different, highly clustered topology with higher reciprocity, suggesting the importance of these neuropeptide systems in the cohesion of the nervous system. While these extrasynaptic networks are separate and non-overlapping with the wired connectome, the hubs of both the wired and wireless networks are interconnected, with multilink motifs showing interaction between the systems at specific points in the network. This suggests that the extrasynaptic networks function both independently–coordinating for example through the monoamine rich-club–and in unison with the synaptic network through multilayer hubs such as RIM and through overrepresented multilink motifs.

The low degree of overlap between the monoamine and synaptic networks occurs not only because many neurons expressing monoamine receptors are not postsynaptic to aminergic neurons, but also because many postsynaptic targets of aminergic neurons to not appear to express monoamine receptors. Some of these synapses could be explained by cotransmission; in particular, tyraminergic and serotonergic neurons also express either cholinergic or glutaminergic markers, and thus classical transmitters could be used in these wired synapses. However, the dopaminergic and octopaminergic neurons of *C*. *elegans* are not known to coexpress any classical neurotransmitter. A second possibility is that these synapses could utilize synaptically-released peptides as neurotransmitters. A third possibility is that the postsynaptic cells might express either an unknown monoamine receptor, or a known one at levels too low to be detected using existing reporters. Finally, it is possible that these putative synapses, which have been identified on the basis of electron micrographs, are not really functional synapses. Further work will be necessary to resolve this puzzling question.

The importance of extrasynaptic neuromodulation to the function of neural circuits is clearly established, for example from work on crustacean stomatogastric circuits [13]. However, systematic attempts to map whole-organism connectomes have focused primarily on chemical synapses, with even gap junctions being difficult to identify using high-throughput electron microscopy approaches [53]. The incorporation of extrasynaptic neuromodulatory interactions, inferred here from gene expression data, adds a large number of new links largely non-overlapping with those of the wired connectome. Although the valence and strength of these inferred neuromodulatory links are largely unknown (information also lacking for much of the synaptic connectome), the monoamine and neuropeptide networks described here nonetheless provide a far more complete picture of potential pathways of communication between different parts of the *C*. *elegans* nervous system.

### Topological properties of monoamine and neuropeptide networks

Although monoamine and neuropeptide signalling both occur extrasynaptically and act on similar timescales, the monoamine and neuropeptide networks show distinct topologies, perhaps reflecting differences in biological function. As noted previously, the monoamine network has a star-like architecture that is qualitatively different to the other network layers. This structure is reflected in the network's high disassortativity and in the low number of recurrent connections. In addition, we observed that the monoamine network contains a rich-club of highly interconnected high-degree releasing neurons, whose members are distinct from (though linked to) the rich-club of the wired connectome. Together, this structure is well-suited to the organisation of collective network dynamics, and is a useful feature given the role of monoamines in signalling physiologically important states relevant to the entire organism, such as food availability.

Despite enormous differences in scale, the monoamine systems of *C*. *elegans* and mammals share a number of common properties suggestive of common network topology. As in *C*. *elegans*, mammalian brains contain a relatively small number of monoamine-releasing neurons that project widely to diverse brain regions; for example, in humans serotonin is produced by less than 100,000 cells in the raphe nuclei, or one millionth of all brain neurons [54]. Moreover, extrasynaptic volume transmission is thought to account for much, if not most, monoamine signalling throughout the mammalian brain [55, 56]. Parallels between monoamine systems in *C*. *elegans* and larger nervous systems are not exact; for example, in *C*. *elegans*, most if not all aminergic neurons appear capable of long-distance signalling, whereas monoamines in larger nervous systems can be restricted by glial diffusion barriers [57]. Nonetheless, mammalian monoamine-releasing neurons, like their *C*. *elegans* counterparts, appear to function as high-degree broadcasting hubs with functionally and spatially diverse targets [54]. Thus, understanding how such hubs act within the context of the completely mapped wired circuitry of *C*. *elegans*, may provide useful insights into the currently unknown structures of multilayer neuronal networks in larger animals.

Although the neuropeptide network has been only partially characterized, the partial network analysed here suggests it may differ in important ways from the other connectome layers, including the monoamine network. In particular, the neuropeptide layer shows strikingly high clustering, even taking into account its high density of connections, and higher reciprocity than the monoamine network. These properties suggest the neuropeptide networks are important for cohesiveness within the nervous system. Multilink analysis also identified differences between the extrasynaptic monoamine and neuropeptide networks. In both cases, a unidirectional extrasynaptic connection coincident with a reciprocal synaptic connection (*motif 10*) was overrepresented in the multiplex connectome. This motif is well-suited to provide feedback between linked nodes, and occurs in several microcircuits implicated in learning and memory. For neuropeptides, a second multilink motif, involving reciprocal neuromodulatory and synaptic connections coincident with a gap junction (*motif 20*) was even more highly overrepresented. This motif occurs in several places in the RMG-centred hub-and-spoke circuit that plays a key role in control of aggregation and arousal. As more neuropeptide systems become characterized, it is reasonable to expect additional examples of this motif will be identified; these may likewise have important computational roles in key neural circuits.

### A prototype for multiplex network analysis

While network theory has occasionally provided novel insights in *C*. *elegans* biology, more often the *C*. *elegans* wired connectome has provided a useful test-bed for validating new network theoretical concepts or their application to larger mammalian brains [10]. In recent years, multilayer complex systems have become an area of intense focus within network science, with a large number of papers dedicated to extending classical network metrics to the multilayer case and to developing new frameworks to understand the dynamical properties of multilayer systems [58].

By definition, multilayer networks contain much more information than simple monoplex networks, leading to significant data-collection challenges. In social networks, for example, large monoplex datasets have been collected describing various types of interactions between people, but these are typically disparate datasets based on different populations. Multiplex datasets combining various edge types into a number of layers are often restricted in size (the number of nodes for which data are collected) or in the choice of edges it is possible to consider (interaction types constrained by data availability) [58].

The multiplex connectome of *C*. *elegans* has the potential to emerge as a gold standard in the study of multilayer networks, much like the wired *C*. *elegans* connectome has for the study of simple monoplex networks over the last 15 years. The synaptic, gap junction, and monoamine layers already represent a relatively reliable and complete mapping of three distinct connection types. The lack of degree-degree correlation between some of these layers suggests that they are not just different facets of one true underlying network (such that each edge is essentially duplicated across all layers). Rather, it suggests that the wired and wireless layers provide distinct channels of communication with differing functional roles. We therefore expect wired and wireless connections to be coupled in functionally relevant 3-node and 4-node motif structures [59, 60], such as (for example) monoamine-based feedback loops or monoamine-regulated wired interactions. The different time-scales on which each of the layers operates are also likely to allow the emergence of interesting dynamical phenomena. Finally, the large number of distinct extrasynaptic interactions offers the scope for a more refined dataset, each aligned to the same complete set of 302 nodes.

### Prospects for complete mapping of multilayer connectomes

How feasible is it to obtain a complete multiplex neuronal connectome? Although the neuropeptide network described here represents only a sample of the total network, the monoamine network already represents a reasonable draft of a complete monoamine connectome. Since expression patterns for amine receptors have been based on reporter coexpression with well-characterized markers, the rate of false positives (i.e. neurons falsely identified as monoamine receptor expressing) is probably very low. In contrast, the false-negative rate (monoamine receptor-expressing cells not included in the network) is almost certainly somewhat higher. In some cases (e.g. *dop-4* and *dop-3* in ASH [27, 61]), reporter transgenes appear to underreport full functional expression domains; in others (e.g. *ser-5*) only a subset of cells expressing a particular reporter have been identified [62]. With recently developed marker strains [60, 63], it should be possible to revisit cell identification and fill in at least some of these missing gaps. In addition, other monoamines (e.g. melatonin [64]) might function as neuromodulators in *C*. *elegans*, and some of the currently uncharacterized orphan receptors in the worm genome [19] might respond to monoamines. Potentially, some of these receptors might be expressed in postsynaptic targets of aminergic neurons (in particular, those of dopaminergic and octopaminergic neurons, which are not known to express classical neurotransmitters). However, the existence of additional monoamine receptor-expressing cells also means that non-synaptic edges are almost certainly undercounted in the network. Thus, the high degree of monoamine releasing hubs–and their importance for intraneuronal signalling outside the wired connectome–is if anything understated by the current findings.

In the future, it should be possible to expand the scope of the multilayer connectome to gain a more complete picture of intraneuronal functional connectivity. Obtaining extrasynaptic connectomes for larger brains, especially those of mammals, will likely be vastly more complicated than for *C*. *elegans*, due not only to the increase in size, but also the existence of additional structural and dynamical properties, such as glial barriers, cellular swelling, and arterial pulsations, all of which dynamically alter extracellular diffusion [65, 66]. In contrast, reanalysis of reporters for monoamine receptors using recently developed reference strains [60, 63] could provide a largely complete monoamine signalling network for *C*. *elegans*. A greater challenge would be to obtain a complete neuropeptide network; this would require comprehensive de-orphanization of neuropeptide GPCRs as well as expression patterns for hundreds of receptor and peptide genes. Additional layers of neuronal connectivity also remain unmapped, such as extrasynaptic signalling by insulin-like peptides, purines, and classical neurotransmitters such as acetylcholine and GABA [67–69]. Obtaining this information, while difficult, is uniquely feasible in *C*. *elegans* given the small size and precise cellular characterisation of its nervous system. Such a comprehensive multilayer connectome could serve as a prototype for understanding how different modes of signalling interact in the context of neuronal circuitry.

## Materials & Methods

### Synaptic & gap junction networks

The synaptic and gap junction networks used in this work were based on the full hermaphrodite *C*. *elegans* connectome, containing all 302 neurons. This network was composed from the somatic connectome of White et al [2], updated and released by the Chklovskii lab [5, 70]; and the pharyngeal network of Albertson and Thomson [3], made available by the Cybernetic *Caenorhabditis elegans* Program (CCeP) (http://ims.dse.ibaraki.ac.jp/ccep/) [71]. The functional classifications referred to in the text (i.e. *sensory neuron*, *interneuron*, *motorneuron*) are based on the classification scheme used in WormAtlas [72]. The gap junction network was modelled as an undirected network with bidirectional electrical synapses; note however that some gap junctions might be rectifying and thus exhibit directionality.

### Monoamine network construction

To map the aminergic signalling networks of *C*. *elegans*, a literature search was first performed to identify genes known to be receptors, transporters or synthetic enzymes of monoamines. A further search was performed to collect cell-level expression data for the monoamine associated genes identified in the previous step. This search was assisted with the curated expression databases of WormBase (Version: WS248; http://www.wormbase.org/) [73] and WormWeb (Version date: 2014-11-16)[74]. A summary of these data is in Supplemental S1–S7 Tables. Neurons expressing multiple receptors for a single monoamine receive a single edge from each sending neuron. Reciprocal connections between nodes are considered as two separate unidirectional connections. Edge lists for individual network are provided in S1 Dataset.

### Neuropeptide network construction

The neuropeptide network was constructed from published expression data for peptides and receptors, using an approach similar to that used for the monoamines. Only those systems were included for which sufficient expression and ligand-receptor interaction data existed in the literature, with interactions being limited to those with biologically plausible peptide-receptor EC_50_ values (Supplemental S8–S10 Tables). In total, 15 neuropeptides and 12 receptors were matched and included in the network. Networks were classified by receptor, allowing a many-to-many relationship between neuropeptides and receptors.

### Neuron identification & microscopy

The expression patterns of the dopamine receptors were determined using the reporter strains DA1646 *lin-15B & lin-15A(n765) X; adEx1646 [lin-15(+) T02E9*.*3(dop-5)*::*GFP]*, BC13771 *dpy-5(e907) I; sEX13771 [rCesC24A8*.*1(dop-6)*::*GFP + pCeh361]*, and FQ78 *wzIs26 [lgc-53*::*gfp; lin-15(+)];lin-15B & lin-15A(n765)* (kindly provided by Niels Ringstad).

The neurons expressing the receptors were identified based on the position and shape of the cell bodies and in most cases co-labelling with other markers. The reporter strains were all crossed with the cholinergic reporter [60] OH13646 *pha-1(e2123) III; him-5(e1490) V; otIs544 [cho-1(fosmid)*::*SL2*::*mCherry*::*H2B + pha-1(+)]* and the glutamatergic reporter [63]OH13645 *pha-1(e2123) III; him-5(e1490) V; otIs518 [eat-4(fosmid)*::*SL2*::*mCherry*::*H2B + pha-1(+)]* (both kindly provided by Oliver Hobert), and dye-filled with DiI using standard procedures. Strains were also crossed to AQ3072 *ljEx540[cat-1*::*mcherry]* and PT2351 *him-5(e1490) V; myEx741 [pdfr-1(3kb)*::*NLS*::*RFP + unc-122*::*GFP]*, which label cells expressing the vesicular monoamine transporter and the PDFR-1 receptor, respectively. When ambiguous, reporter strains were crossed with additional strains, as listed below.

Reporter expression in individual neurons was confirmed with the following crosses:

For *dop-5*:

AIM and ADF were confirmed based on coexpression with *cat-1*. URX, PVC, RIF, RIB, AIY, M5, and DVA were identified based on position and coexpression with *cho-1*[60]. MI, DVC, ASE (previously identified in [75]) and ADA were confirmed based on position and coexpression with *eat-4* [63]. ASI, PHA and PHB were confirmed based on costaining with DiI. PVT, RMG and BDU were identified based on cell body position and shape alone.

For *dop-6*:

RIH and ADF were confirmed based on coexpression with *cat-1*[24]. ASI and PHA were confirmed based on costaining with DiI. AQ3499 *ljEx805 [sra-6*::*mcherry + PRF4]* was used to confirm expression in PVQ. AQ3682 *ljEx921[flp-8*::*mcherry + unc-122*::*gfp]* was used to confirm expression in URX and AUA. IL2, RIB, RMD and URA were identified based on position and coexpression with *cho-1*. AVF was identified based on position and failure to coexpress *eat-4* and *cho-1*. RID was identified based on position relative to URX and morphology.

For *lgc-53*:

AIM was confirmed based on coexpression of *cat-1*. AVF was confirmed based on coexpression with *pdfr-1* and failure to coexpress *eat-4* and *cho-1*. URY was confirmed based on position, coexpression with *eat-4*, and lack of coexpression with *ocr-4*. AQ3526 *ljEx822 [klp-6*::*mcherry + pRF4]* was used to confirm IL2 expression. AQ3535 *ljEx828 [unc-4*::*mcherry + pRF4]* was used to confirm VA expression. FLP was confirmed based on position, morphology, and coexpression with *eat-4*. HSN, CAN and PVD expression were identified based on position and morphology.

### Microscopy

Strains were examined using a Zeiss Axioskop. Images were taken using a Zeiss LSM780 confocal microscope. Worms were immobilized on 3% agarose pads with 2.5mM levamisole. Image stacks were acquired with the Zen 2010 software and processed with Image J.

### Topological network measures

Edge counts, adjacency matrices and reducibility clusters were all computed using binary directed versions of the networks. The same networks, excluding self-connections (i.e. setting all diagonal elements to 0), were used to compute all other measures.

Network measures are compared to 100 null model networks (shown in the boxplots) generated using the degree-preserving edge swap procedure. This is performed by selecting a pair of edges (*A*→*B*) (*C*→*D*) and swapping them to give (*A*→*D*)(*C*→*B*). If the resulting edges already exist in the network, another pair of edges is selected instead. Each edge was swapped 10 times to ensure full randomisation. To compute the multilink motif *z*-scores, the null model was constructed by randomizing each layer independently.

To identify neurons with high-participation in all of the network layers, the normalized degree-rank product was used. This is computed by ranking neurons in each network layer by their degree in descending order, and scaling to the range [0, 1]. The product is then taken of the ranked degrees in each layer. Thus, if a neuron had the highest degree in each of the network layers, it would have a degree product of 1.

### Clustering coefficient

The measure of clustering described here is the global clustering, also known as *transitivity*, given in [76–78], which measures the ratio of triangles to triples (where a triple is a single node with edges running to an unordered pair of others, and a triangle is a fully-connected triple). For a directed network, this is equivalent to:

$$T=\frac{\sum_{i\in N}t_{i}}{\sum_{i\in N}\left[(k_{i}^{out}+k_{i}^{in})(k_{i}^{out}+k_{i}^{in}-1)-2\sum_{j\in N}A_{ij}A_{ji}\right]}$$

where *A* is the adjacency matrix, *N* is the number of nodes, *k*^*out*^ and *k*^*in*^ are the out-degree and in-degree, and *t*_*i*_ is the number of triangles around a node:

$$t_{i}=\frac{1}{2}\sum_{j,h\in N}{(A}_{ij}+A_{ji})\;(A_{ih}+A_{hi})(A_{jh}+A_{hj})$$

### Characteristic path length

To obtain the characteristic path length of a network, the geodesic (i.e. minimum) distance, *d*, between each pair of nodes *i*, *j*, is first computed:

$$d_{ij}=\sum_{A_{uv}\in g(i,j)}A_{uv}$$

where *g(i*,*j)* returns the geodesic path between nodes *i* and *j*. The characteristic path length is then given:

$$L=\frac{1}{n}\sum_{i\in N}\frac{\sum_{j\in N,i\neq j}d_{ij}}{n-1}$$

### Modularity

The modularity *Q* is determined by first subdividing the network into non-overlapping modules *c* to maximise within-module connectivity and minimise between-module connectivity [79]. The modularity then gives the proportion of edges that connect to nodes within the same module:

$$Q=\frac{1}{M}\sum_{i,j\in N}\left(A_{ij}-\frac{k_{i}^{in}k_{j}^{out}}{M}\right)\delta (c_{i},c_{j})$$

where *c*_*i*_, *c*_*j*_ are the modules respectively containing nodes *i*, *j*; *M* is the number of edges, and *δ* is the Kronecker delta function:

$$\delta (x,y)=\left\{ \begin{array}{c} 1\;\text{if}\;x=y\\ 0\;\text{if}\;x\neq y \end{array}\right.$$

### Assortativity

The assortativity of a network is the correlation between the degrees of nodes on either side of a link. This is given by Newman [80] as:

$$R=\frac{M^{-1}\sum_{ij\in E}k_{i}^{out}k_{j}^{in}-{\left[M^{-1}\sum_{ij\in E}\frac{1}{2}(k_{i}^{out}+k_{j}^{in})\right]}^{2}}{M^{-1}\sum_{ij\in E}\frac{1}{2}(\left[{\left(k_{i}^{out}\right)}^{2}+{\left(k_{j}^{in}\right)}^{2}\right])-{\left[M^{-1}\sum_{ij\in E}\frac{1}{2}(k_{i}^{out}+k_{j}^{in})\right]}^{2}}$$

### Reducibility

Structural reducibility measures the uniqueness of layers by comparing the relative Von Neumann entropies. The larger the relative entropy, the more distinguishable the layer. Formally, the Von Neumann entropy for a layer is given:

$$H=-\sum_{i}^{N}\lambda_{i}^{\left[\alpha \right]}\log_{2}\lambda_{i}^{\left[\alpha \right]}$$

where $\lambda_{i}^{\left[\alpha \right]}$ are the eigenvalues of the Laplacian matrix associated to layer *A*^[*α*]^. To visualise layer similarity, hierarchical clustering was performed using the Jensen-Shannon distance [28] and the Ward hierarchical clustering method [81].

### Reciprocity

Reciprocity is the fraction of reciprocal edges in the network:

$$r=\frac{\left|E^{↔}\right|}{M}$$

where *M* is the number of edges, and |*E*^↔^| is the number of reciprocal edges:

$$|E^{↔}|=\sum_{i\neq j}A_{ij}A_{ji}$$

### Rich-club coefficient

The rich-club phenomenon is the tendency for high-degree nodes in a network to form highly-interconnected communities [40, 82]. Such communities can be identified by creating subnetworks for each degree level *k*, where nodes with a degree ≤ *k* are removed, and computing the rich-club coefficient Φ(*k*) for each subnetwork. This is the ratio of remaining connections *M*_*k*_ to the maximum possible number of connections. For a directed network with no self-connections, where *N*_*k*_ is the number of remaining nodes, this is given by:

$$\Phi (k)=\frac{M_{k}}{N_{k}(N_{k}-1)}$$

Thus, a fully-connected subnetwork at a given degree *k* has a rich-club coefficient Φ(*k*) = 1. To normalise the rich-club coefficient, we computed the average values for 100 random networks ⟨Φ_*random*_(*k*)⟩:

$$\Phi_{norm}(k)=\frac{\Phi (k)}{\left\langle \Phi_{random}(k)\right\rangle }$$

We used the same threshold previously used in determining the wired rich-club of *C*. *elegans* [11], defining a rich-club to exist where Φ_*norm*_(*k*) ≥ 1 + 1*σ*, where *σ* is the Standard Deviation of Φ_*random*_(*k*).

### Multilink motifs

Multilink motif analysis considers the full range of possible link combinations that can exist between any two nodes across all layers of a network, and is based on the concept of multilink as described in [47, 83, 84]. Due to the conceptual and structural similarity between monoamine layers (see *reducibility*), we limited our analysis to three layers: synaptic, gap junction, and monoamine (see SI for neuropeptides), giving a total of 20 possible multilink motifs. Instances of each motif were recorded by simultaneously traversing the three network layers. This was also conducted for 100 randomized three-layer networks, generated by rewiring each of the real networks individually using the same randomisation procedure described above. These random networks were used to calculate motif *z*-scores and *p*-values for the actual network.

### Software

Network measures were computed in MATLAB (v8.5, The MathWorks Inc., Natick, MA) using the Brain Connectivity Toolbox [77] and MATLAB/Octave Networks Toolbox [85]. Reducibility analysis, clustering, and multilayer plots were computed in MuxViz [86]. Reducibility is based on the algorithm described in [28], and layer similarity was visualized using the Ward hierarchical clustering method [81]. Hive plots were generated using the custom hiveplotter function written in Python (Python Software Foundation. Python Language Reference, v3.5). 3-node network motifs were computed using FANMOD [87]. Additional network visualisations were created using Cytoscape [88] and Dia (https://wiki.gnome.org/Apps/Dia/).

## Supporting Information

S1 Fig Topological properties of additional C. elegans extrasynaptic networks. (A) Multilayer expansion of monoamine subnetworks using the larger (*dop-5/6*-containing) dopamine network. Node positions are the same in all layers. (B-F) Comparison of network metrics for the dopamine (DA, with/without *dop-5/6)*, serotonin (5-HT), tyramine (TA), octopamine (OA) or aggregate monoamine including *dop-5/6* networks. Plots show the observed values (filled squares) and expected values for 100 rewired networks preserving degree distribution (boxplots).
(TIFF)

S2 Fig Three-neuron motif analysis for monoamine networks. Directed 3-node motifs for the monoamine networks, showing all 13 possible combinations with no unconnected nodes. Z-scores show the level of over- or under- representation for each motif, and were computed relative to a sample of a 100 random networks generated using the degree-persevering randomisation procedure with 10 swaps per edge. Motif enumeration was performed using the FANMOD algorithm (see Methods).
(TIFF)

S3 Fig Additional multilink analysis. Shown are overrepresented and underrepresented multilink motifs for 3-layer networks consisting of synaptic, gap junction and indicate extrasynaptic layers. (A) Multilink motif IDs. These correspond to all possible configurations of links between two neurons allowing for: no connection of a given type (dotted line), directed extrasynaptic monoamine links (Ext, represented as arrows on the top), bidirectional gap junctions (represented as bars in the middle) and synapses (represented as inverted arrowheads on the bottom line). (B-E) Motif *z*-scores for aggregate monoamines including *dop-5/6* (B), neuropeptide (C), dopamine including *dop-5/6*, (D) or tyramine (E) 3-layer multilink. Over-represented motifs are represented by red upward-pointing triangles. Under-represented motifs are represented by blue downward-pointing triangles. Non-significant motifs are shown by black squares. Values for randomized null model networks are shown as grey crosses. Asterisks report the significance level using the z-test, with Bonferroni-adjusted p-values: * indicates *p* ≤ 0.05; ** indicates *p* ≤ 0.01; *** indicates *p* ≤ 0.001; **** indicates *p* ≤ 0.0001. Observed and expected multilink frequencies are in Table 5. Examples of monoamine *motif 10* are listed in Table 6.
(TIFF)

S1 Dataset Included are edge lists for monoamine and neuropeptide networks (ZIP)

S1 Table Serotonin (5-HT) expressing cells. Cells with weak or conditional expression (not included in the network) are marked †
(DOCX)

S2 Table Dopamine (DA) expressing cells (DOCX)

S3 Table Octopamine (OA) & tyramine (TA) expressing cells. ⋆RIC is excluded from the TA network due to co-expression of *tbh-1* which converts TA to OA
(DOCX)

S4 Table Serotonin (5-HT) receptor expression patterns (DOCX)

S5 Table Octopamine (OA) receptor expression patterns (DOCX)

S6 Table Dopamine (DA) receptor expression patterns (DOCX)

S7 Table Tyramine (TA) receptor expression patterns (DOCX)

S8 Table Neuropeptide expression patterns (DOCX)

S9 Table Neuropeptide receptor expression patterns (DOCX)

S10 Table Neuropeptide receptor-ligand binding. ⋆No EC_50_ value reported for NPR- 11/NLP-1; strong biological activity seen in the micromolar range
(DOCX)

S11 Table Examples of octopamine multilink *motifs 9* and *11*. List of neurons connected by *motif 9* (i.e. unidirectional OA link and synapse in reverse direction) or *motif 11* (shaded, unidirectional OA link coincident with gap junction)
(DOCX)

S12 Table 4-layer (syn, gap, MA, NP) normalized degree product (DOCX)

S13 Table Multilink motif frequencies for the neuropeptide, synaptic and gap junction layers. Motif IDs correspond to those depicted in Figs 7 & S3.
(DOCX)

S14 Table Examples of neuropeptide multilink *motif 20*. List of neurons connected by *motif 20* (i.e. reciprocal NP link, gap junction, and reciprocal synapses)
(DOCX)

S1 References References for Supplemental Tables (DOCX)
