# Supplementary figures and images for: The Multilayer Connectome of Caenorhabditis elegans
Source: PLoS Comput Biol. 2016 Dec 16;12(12):e1005283. doi: 10.1371/journal.pcbi.1005283 (PMC5215746; doi:10.1371/journal.pcbi.1005283)

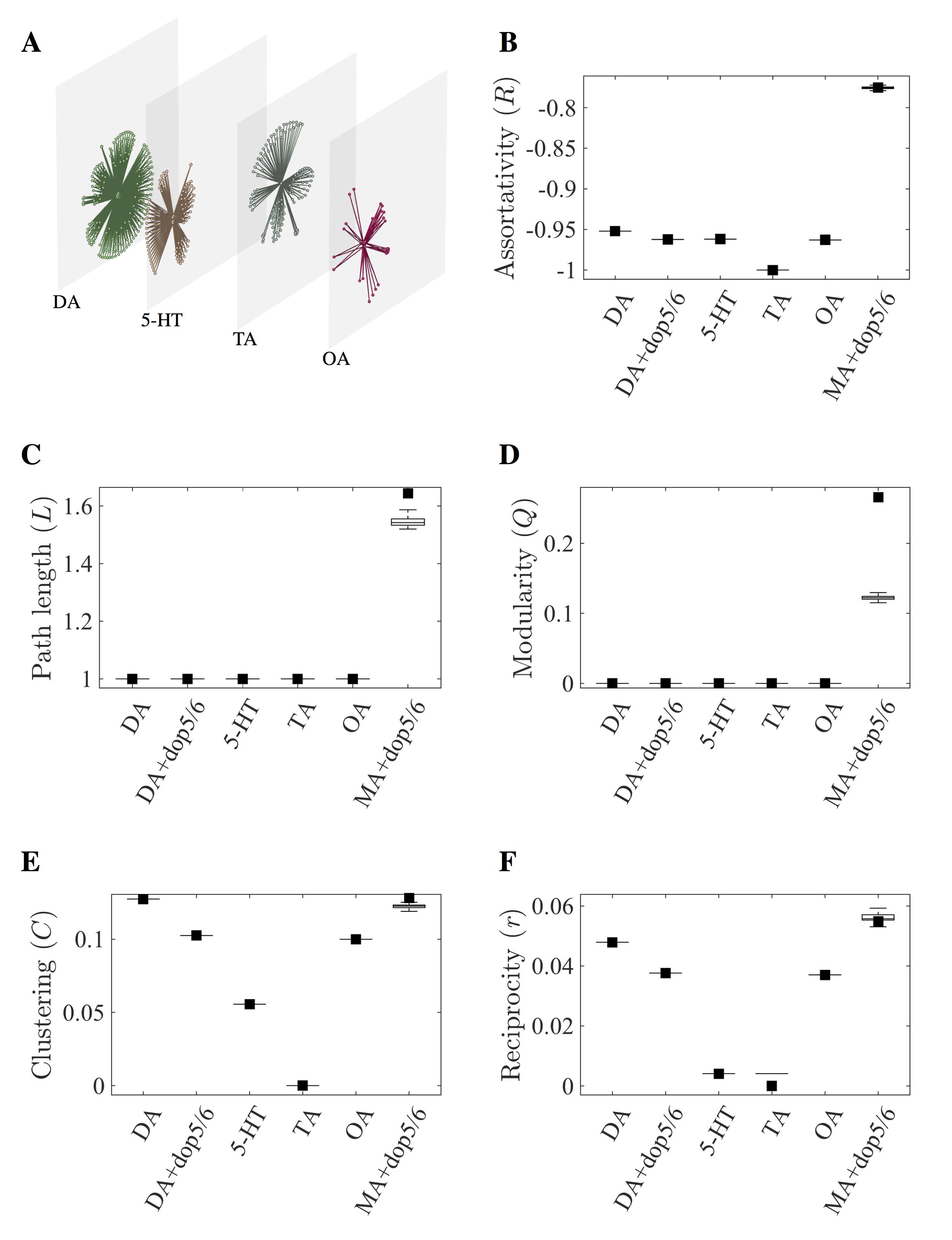

Supplement: S1 Fig — (A) Multilayer expansion of monoamine subnetworks using the larger (dop-5/6-containing) dopamine network. Node positions are the same in all layers. (B-F) Comparison of network metrics for the dopamine (DA, with/without dop-5/6), serotonin (5-HT), tyramine (TA), octopamine (OA) or aggregate monoamine including dop-5/6 networks. Plots show the observed values (filled squares) and expected values for 100 rewired networks preserving degree distribution (boxplots). (TIFF) [file pcbi.1005283.s001.tiff]

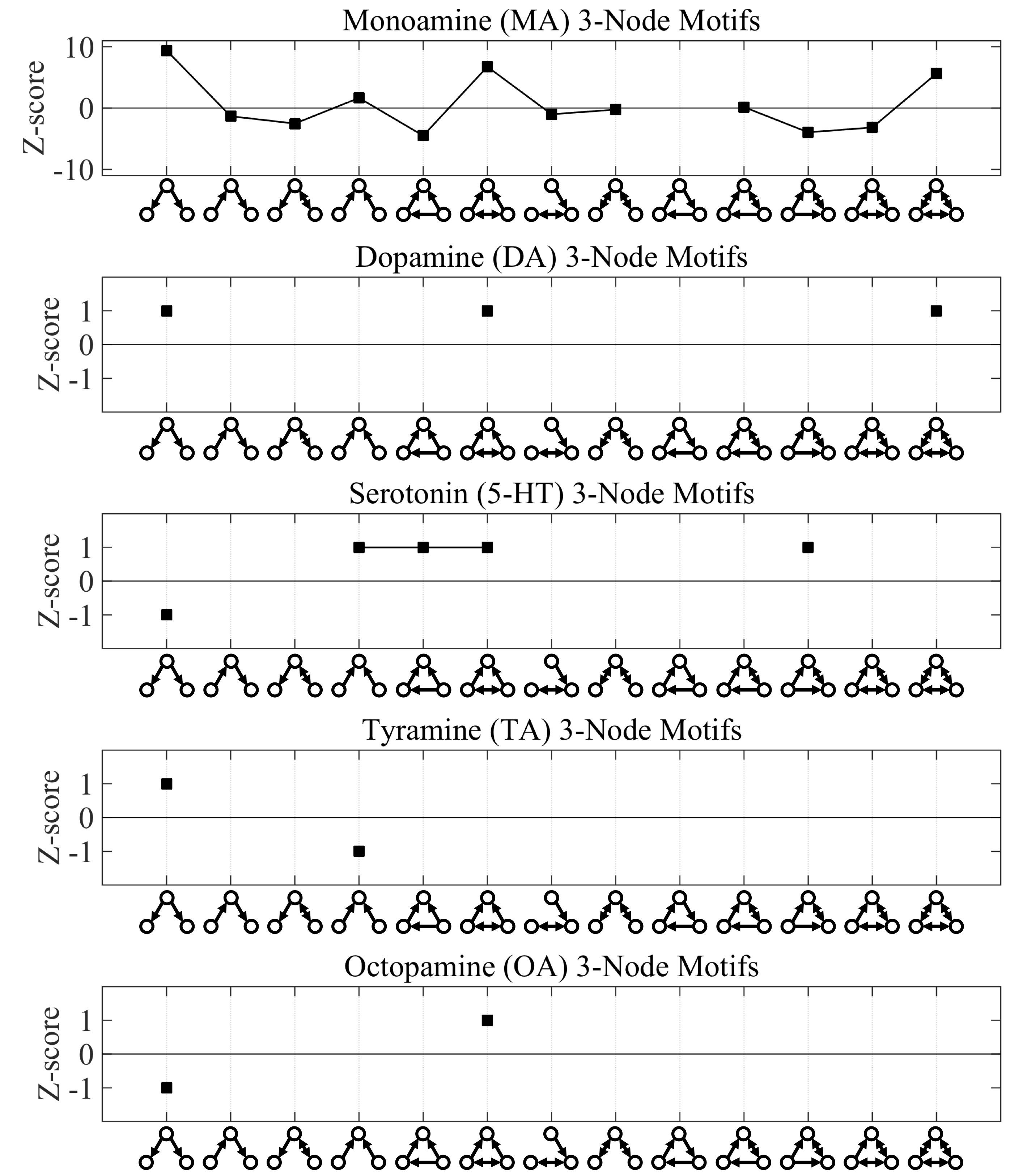

Supplement: S2 Fig — Directed 3-node motifs for the monoamine networks, showing all 13 possible combinations with no unconnected nodes. Z-scores show the level of over- or under- representation for each motif, and were computed relative to a sample of a 100 random networks generated using the degree-persevering randomisation procedure with 10 swaps per edge. Motif enumeration was performed using the FANMOD algorithm (see Methods). (TIFF) [file pcbi.1005283.s002.tiff]

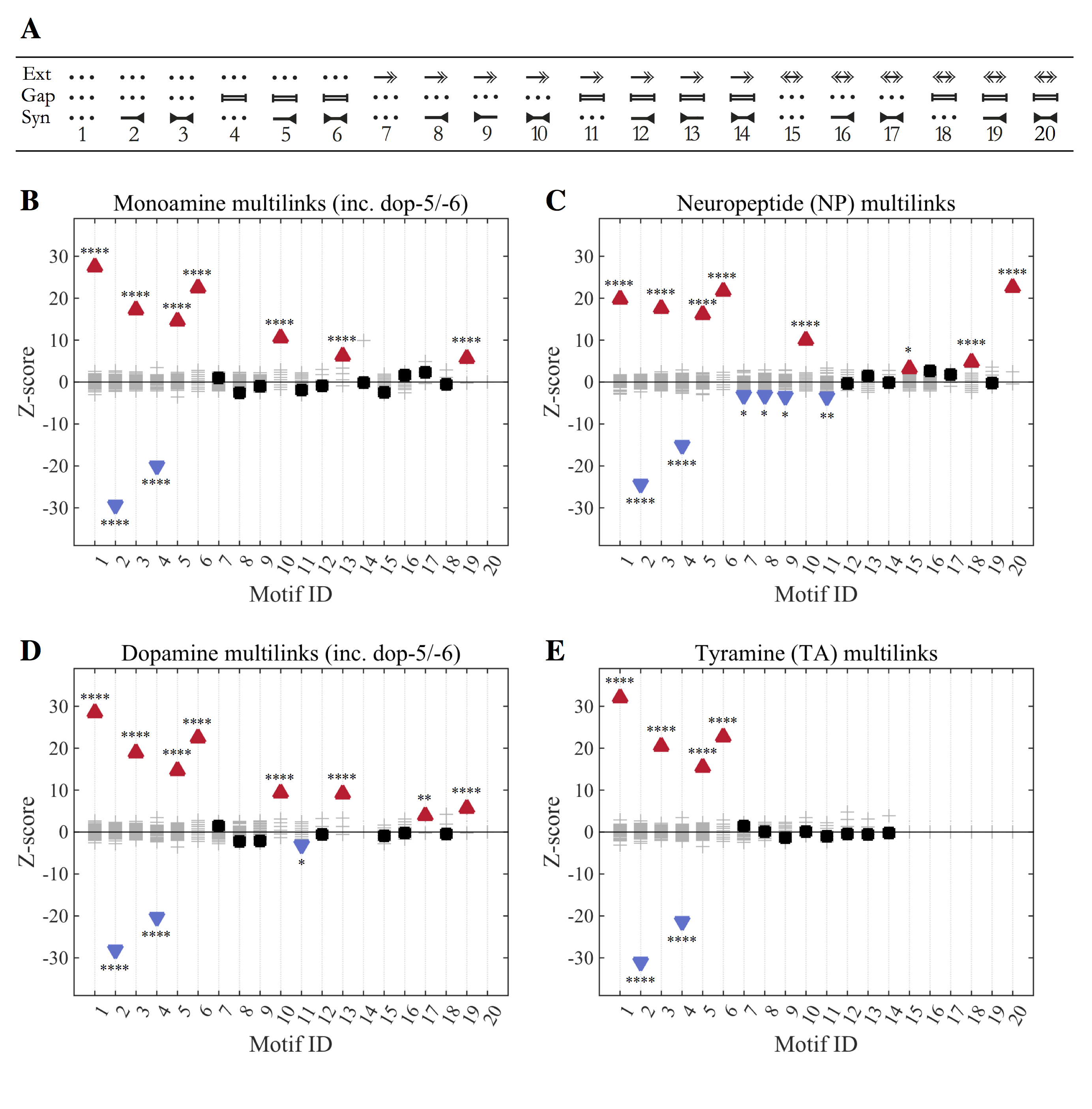

Supplement: S3 Fig — Shown are overrepresented and underrepresented multilink motifs for 3-layer networks consisting of synaptic, gap junction and indicate extrasynaptic layers. (A) Multilink motif IDs. These correspond to all possible configurations of links between two neurons allowing for: no connection of a given type (dotted line), directed extrasynaptic monoamine links (Ext, represented as arrows on the top), bidirectional gap junctions (represented as bars in the middle) and synapses (represented as inverted arrowheads on the bottom line). (B-E) Motif z-scores for aggregate monoamines including dop-5/6 (B), neuropeptide (C), dopamine including dop-5/6, (D) or tyramine (E) 3-layer multilink. Over-represented motifs are represented by red upward-pointing triangles. Under-represented motifs are represented by blue downward-pointing triangles. Non-significant motifs are shown by black squares. Values for randomized null model networks are shown as grey crosses. Asterisks report the significance level using the z-test, with Bonferroni-adjusted p-values: * indicates p ≤ 0.05; ** indicates p ≤ 0.01; *** indicates p ≤ 0.001; **** indicates p ≤ 0.0001. Observed and expected multilink frequencies are in Table 5. Examples of monoamine motif 10 are listed in Table 6. (TIFF) [file pcbi.1005283.s003.tiff]
