# Supplementary material for: The Multilayer Connectome of Caenorhabditis elegans
Source: PLoS Comput Biol. 2016 Dec 16;12(12):e1005283. doi: 10.1371/journal.pcbi.1005283 (PMC5215746; doi:10.1371/journal.pcbi.1005283)
Supplement: S1 Table — Cells with weak or conditional expression (not included in the network) are marked † (DOCX) [file pcbi.1005283.s005.docx]

| **Marker** | **WormBase ID** | **Neurons** | **Reference** |
| --- | --- | --- | --- |
| *tph-1* | Expr959 | RIH†, AIM†, ADF, NSM, HSN | [[1](#_ENREF_1)] |
|  | Expr12176 | ASG† | [[2](#_ENREF_2)] |
| *mod-5* | Expr9350 | AIM†, NSM, ADF, RIH† | [[3](#_ENREF_3)] |
