# Supplementary material for: The Multilayer Connectome of Caenorhabditis elegans
Source: PLoS Comput Biol. 2016 Dec 16;12(12):e1005283. doi: 10.1371/journal.pcbi.1005283 (PMC5215746; doi:10.1371/journal.pcbi.1005283)
Supplement: S2 Table — (DOCX) [file pcbi.1005283.s006.docx]

| **Marker** | | **WormBase ID** | **Neurons** | **Reference** |
| --- | --- | --- | --- | --- |
| *cat-2* | Expr2619 | | ADE, PDE, CEP | [[4](#_ENREF_4)] |
| *dat-1* | Expr8327 | | ADE, PDE, CEP | [[5](#_ENREF_5)] |
