# Supplementary material for: The Multilayer Connectome of Caenorhabditis elegans
Source: PLoS Comput Biol. 2016 Dec 16;12(12):e1005283. doi: 10.1371/journal.pcbi.1005283 (PMC5215746; doi:10.1371/journal.pcbi.1005283)
Supplement: S3 Table — ⋆RIC is excluded from the TA network due to co-expression of tbh-1 which converts TA to OA (DOCX) [file pcbi.1005283.s007.docx]

| **Marker** | **WormBase ID** | **Neurons** | **Reference** |
| --- | --- | --- | --- |
| **Octopamine**  *tbh-1* | Expr3721 | RIC | [[6](#_ENREF_6)] |
| **Tyramine**  *tdc-1* | Expr3722 | RIM, RIC⋆ | [[6](#_ENREF_6)] |
