# Supplementary material for: The Multilayer Connectome of Caenorhabditis elegans
Source: PLoS Comput Biol. 2016 Dec 16;12(12):e1005283. doi: 10.1371/journal.pcbi.1005283 (PMC5215746; doi:10.1371/journal.pcbi.1005283)
Supplement: S4 Table — (DOCX) [file pcbi.1005283.s008.docx]

| **Marker** | **WormBase ID** | **Neurons** | **Reference** |
| --- | --- | --- | --- |
| *ser-1* | Expr7825 | RIA, RIC, PVT, DVC, URY | [[7](#_ENREF_7)] |
|  | Expr8282 | PVQ | [[8](#_ENREF_8)] |
|  | Expr3962 | RMD, RMF, RMH | [[9](#_ENREF_9)] |
| *ser-4* | Expr2710 | RIB, PVT, DVC, DVA, RIS | [[10](#_ENREF_10)] |
|  | Expr10554 | AIB, NSM | [[11](#_ENREF_11)] |
|  | N/A | M1, RIM | [[12](#_ENREF_12)] |
| *ser-5* | Expr12174 | ASH, AWB | [[13](#_ENREF_13)] |
|  | Expr12172 | AVJ | [[14](#_ENREF_14)] |
| *ser-7* | Expr3759 | MC, M2, M3, M4, M5, I2, I3, I4, I6 | [[15](#_ENREF_15)] |
| *mod-1* | Expr10023 | RIM, RID, RIC, AIZ, AIY, AIB, AIA | [[16](#_ENREF_16)] |
|  | Expr10553 | RME, DD, VD | [[11](#_ENREF_11)] |
