# Supplementary material for: The Multilayer Connectome of Caenorhabditis elegans
Source: PLoS Comput Biol. 2016 Dec 16;12(12):e1005283. doi: 10.1371/journal.pcbi.1005283 (PMC5215746; doi:10.1371/journal.pcbi.1005283)
Supplement: S5 Table — (DOCX) [file pcbi.1005283.s009.docx]

| **Marker** | **WormBase ID** | **Neurons** | **Reference** |
| --- | --- | --- | --- |
| *octr-1* | Expr7846 | ASH, ASI, AIY, ADE, CEP | [[17](#_ENREF_17)] |
| *ser-3* | Expr8275 | PVQ, PHB, PHA, SIA | [[18](#_ENREF_18)] |
|  | Expr10640 | ASH | [[19](#_ENREF_19)] |
| *ser-6* | Expr10641 | AWB, ASI, ADL | [[19](#_ENREF_19)] |
|  | Expr11709 | RIC, SIA | [[20](#_ENREF_20)] |
