# Supplementary material for: The Multilayer Connectome of Caenorhabditis elegans
Source: PLoS Comput Biol. 2016 Dec 16;12(12):e1005283. doi: 10.1371/journal.pcbi.1005283 (PMC5215746; doi:10.1371/journal.pcbi.1005283)
Supplement: S6 Table — (DOCX) [file pcbi.1005283.s010.docx]

| **Marker** | **WormBase ID** | **Neurons** | **Reference** |
| --- | --- | --- | --- |
| *dop-1* | Expr2882 | AUA, RIM, ALM, RIB, PLM, PHC | [[21](#_ENREF_21)] |
|  | Expr2708 | AVM, ALN, PVQ, PLN, RIS | [[10](#_ENREF_10)] |
|  | Expr3047 | PVD, VA, VB, AS, DA, DB | [[22](#_ENREF_22)] |
| *dop-2* | Expr2618 | ADE, PDE, CEP | [[4](#_ENREF_4)] |
|  | Expr2709 | RID, RIA, PDA, SIB, SIA | [[10](#_ENREF_10)] |
| *dop-3* | Expr3048 | PVD, VA, VB, AS, DA, DB, DD, VD | [[22](#_ENREF_22)] |
|  | Expr7939 | ASE | [[23](#_ENREF_23)] |
|  | Expr8667 | RIC, SIA | [[24](#_ENREF_24)] |
|  | Expr11452 | NSM | [[25](#_ENREF_25)] |
|  | Expr12177 | ASK | [[26](#_ENREF_26)] |
| *dop-4* | Expr3687 | AVL, ASG, PQR, I2, I1, CAN | [[27](#_ENREF_27)] |
| *dop-5* | Expr7939 | ASE | [[23](#_ENREF_23)] |
|  | N/A | MI, M5, BDU, RIB, PHA, PHB, DVA, AIM, ADA, DVC, ASI, RMG, PVC, RIF, URX, AIY, PVT | N/A |
| *dop-6* | Expr11993 | OLL | [[28](#_ENREF_28)] |
|  | N/A | AUA, RID, RMD, RIB, ASI, PHA, IL2, PVQ, URA, AVF, ADF, RIH, URX | this study |
| *lgc-53* | N/A | HSN, PVD, CAN, IL2, VA, AIM, FLP, AVF, URY | this study |
