# Supplementary material for: The Multilayer Connectome of Caenorhabditis elegans
Source: PLoS Comput Biol. 2016 Dec 16;12(12):e1005283. doi: 10.1371/journal.pcbi.1005283 (PMC5215746; doi:10.1371/journal.pcbi.1005283)
Supplement: S7 Table — (DOCX) [file pcbi.1005283.s011.docx]

| **Marker** | **WormBase ID** | **Neurons** | **Reference** |
| --- | --- | --- | --- |
| *ser-2* | Expr2707 | BDU, AVH, AUA, ALN, RID, RIC, AIZ, RIA, AIY, PVT, PVD, PVC, OLL, NSM, LUA, DVA, DA09, CAN, SIA, SDQ, SAB, RME | [[10](#_ENREF_10)] |
|  | Expr3206 | PVD | [[29](#_ENREF_29)] |
|  | Expr10758 | VD | [[30](#_ENREF_30)] |
| *tyra-2* | Expr3415 | ASI, ASH, ASG, ASE, ALM, PVD, NSM, MC, CAN | [[31](#_ENREF_31)] |
| *tyra-3* | Expr11003 | BAG, AWC, AUA, ASK, AIM, AFD, ADL, OLQ, CEP, SDQ | [[32](#_ENREF_32)] |
|  | Expr6415 | PVT | [[33](#_ENREF_33)] |
|  | Expr12173 | ADE | [[17](#_ENREF_17)] |
| *lgc-55* | Expr8613 | AVB, ALN, IL1, HSN, SMD, SDQ, RMD | [[34](#_ENREF_34)] |
|  | Expr8997 | AVM, ALM | [[35](#_ENREF_35)] |
