# Supplementary material for: The Multilayer Connectome of Caenorhabditis elegans
Source: PLoS Comput Biol. 2016 Dec 16;12(12):e1005283. doi: 10.1371/journal.pcbi.1005283 (PMC5215746; doi:10.1371/journal.pcbi.1005283)
Supplement: S8 Table — (DOCX) [file pcbi.1005283.s012.docx]

| Receptor | WBID | Neurons | Reference |
| --- | --- | --- | --- |
| *flp-1* | Expr3003 | AVK, AVE, AVA, RIG, AIY, AIA, M5, RMG | [[36](#_ENREF_36)] |
| *flp-4* | Expr3006 | AWC, AVM, ASEL, ADL, PVD, PHB, PHA, NSM, I5, I6, FLP | [[36](#_ENREF_36)] |
| *flp-5* | Expr3007 | ASE, PVT, M4, I4, I2 RMG | [[36](#_ENREF_36)] |
| *flp-10* | Expr3011 | AIM, ASI, AUA, BAG, BDU, DVB, PQR, PVR, URX | [[36](#_ENREF_36)] |
| *flp-13* | Expr3014 | ASE, ASG, ASK, BAG, DD, I5, M3, M5 | [[36](#_ENREF_36)] |
|  | Expr12005 | ALA | [[37](#_ENREF_37)] |
| *flp-15* | Expr3015 | PHA, I2 | [[37](#_ENREF_37)] |
| *flp-17* | Expr3016 | BAG, M5 | [[36](#_ENREF_36)] |
| *flp-18* | Expr3017 | AVA, AIY, RIG, RIM, M2, M3 | [[36](#_ENREF_36)] |
| *flp-21* | Expr3020 | ASI, ASH, ASE, ADL, MC, M4, FLP, URA | [[36](#_ENREF_36)] |
|  | Expr12181 | RMG, ASJ, URX, M2, ASK, ASG, ADF | [[38](#_ENREF_38)] |
| *nlp-1* | Expr1686 | ASI, AWC, PHB, BDU | [[39](#_ENREF_39)] |
|  | Marker88 | HSN | [[40](#_ENREF_40)] |
| *nlp-12* | Expr8057 | DVA | [[41](#_ENREF_41)] |
| *ntc-1* | Expr11371 | AVK, RIC, AIZ, AFD, NSM, M5, DVA, DD, VD, VC | [[42](#_ENREF_42)] |
|  | Expr11368 | ASG | [[43](#_ENREF_43)] |
| *pdf-1* | Expr11002 | AVB, ASK, AIM, AFD, PVT, PVP, PVN, LUA, SIA, SAA, RMG | [[44](#_ENREF_44)] |
|  | Expr9958 | ASI, RID, ADA, ADE, PQR, PHB, PHA, RME | [[41](#_ENREF_41)] |
| *pdf-2 / nlp-3* | Expr9959 | BDU, AVG, AVD, RIM, AQR, RID, AIM, PVT, PVP, PQR, PHB, PHA, RIS | [[41](#_ENREF_41)] |
| *nlp-24* | Expr1717 | ASI | [[39](#_ENREF_39)] |
