# Supplementary material for: The Multilayer Connectome of Caenorhabditis elegans
Source: PLoS Comput Biol. 2016 Dec 16;12(12):e1005283. doi: 10.1371/journal.pcbi.1005283 (PMC5215746; doi:10.1371/journal.pcbi.1005283)
Supplement: S9 Table — (DOCX) [file pcbi.1005283.s013.docx]

| **Receptor** | **WBID** | **Neurons** | **Reference** |
| --- | --- | --- | --- |
| *npr-1* | Expr2257 | AUA, ASH, ASG, ASE, AQR, RIG, PQR, PHB, PHA, OLQ, IL2L, IL2R, URX, SMBDL, SMBDR, RMG, RIV, DD, VD, M3, SAADL, SAADR, SDQ | [[45](#_ENREF_45)] |
| *npr-2* | Expr12242 | ADF, AIZ, ASH, FLP, OLQ, PVD, PVQ, SAB | [[46](#_ENREF_46)] |
| *npr-3* | Expr2766 | AS, DA, DB, VA, VB | [[47](#_ENREF_47)] |
| *npr-4* | Expr8975 | BDU, BAG, AVA, PQR, RIV | [[48](#_ENREF_48)] |
| *npr-5* | Expr8976 | AWB, AWA, AUA, ASK, ASJ, ASI, ASG, ASE, AIA, ADF, PHB, PHA, IL2 | [[48](#_ENREF_48)] |
| *npr-11* | Expr12179 | AIA, AIY | [[49](#_ENREF_49)] |
| *frpr-4* | N/A | RIA, PVM, AVE, I1, DVA | [[37](#_ENREF_37)] |
| *npr-17* | Expr12182 | AVG, ASI, PVP, PVQ, PQR | [[50](#_ENREF_50)] |
| *ckr-2* | Expr10065 | AIY | [[51](#_ENREF_51)] |
|  | Expr12178 | AS, DA, DB, VA, VB | [[52](#_ENREF_52)] |
| *ntr-1* | Expr11372 | ASH, RIC, ADL, ADF, PVW, PVR, PVQ, I2 | [[42](#_ENREF_42)] |
|  | Expr11369 | BDU, ASE, PQR | [[43](#_ENREF_43)] |
| *egl-6* | Expr8338 | HSN, DVA, SDQ | [[53](#_ENREF_53)] |
| *pdfr-1* | Expr10592 | AVM, AVD, RIF, ALM, PVW, PVQ, PVM, PVC, PQR, PLM, PHA, OLL, DB2, URY, URX, RME, AVF | [[44](#_ENREF_44)] |
|  | Expr8177 | PHB, OLQ, I1 FLP | [[41](#_ENREF_41)] |
