# Supplementary material for: The Multilayer Connectome of Caenorhabditis elegans
Source: PLoS Comput Biol. 2016 Dec 16;12(12):e1005283. doi: 10.1371/journal.pcbi.1005283 (PMC5215746; doi:10.1371/journal.pcbi.1005283)
Supplement: S10 Table — ⋆No EC50 value reported for NPR- 11/NLP-1; strong biological activity seen in the micromolar range (DOCX) [file pcbi.1005283.s014.docx]

| **Receptor** | **Ligand** | **EC_50_** | **Reference** |
| --- | --- | --- | --- |
| **NPY(npr) / RFamide receptor group** | | |  |
| NPR-1 | FLP-18 | ~ 100 nM | [[54](#_ENREF_54)] |
|  | FLP-21 | 2.5 nM | [[55](#_ENREF_55)] |
| NPR-2 | FLP-21 | 34.4 nM | [[56](#_ENREF_56)] |
| NPR-3 | FLP-15 | 162-599 nM | [[57](#_ENREF_57)] |
| NPR-4 | FLP-1 | 0.4-9 μM | [[58](#_ENREF_58)] |
|  | FLP-4 | 5-80 nM | [[58](#_ENREF_58)] |
|  | FLP-18 | 5 nM-1.2 μM | [[48](#_ENREF_48)] |
| NPR-5 | FLP-18 | 13.3-117.2 nM | [[59](#_ENREF_59)] |
|  | FLP-21 | 267 nM | [[59](#_ENREF_59)] |
| NPR-11 | FLP-1 | 1-8 μM | [[58](#_ENREF_58)] |
|  | FLP-5 | 1-8 μM | [[58](#_ENREF_58)] |
|  | FLP-18 | 180-800 nM | [[58](#_ENREF_58)] |
|  | FLP-21 | 1-10 nM | [[58](#_ENREF_58)] |
|  | NLP-1 | 1-100 μM? | [[49](#_ENREF_49)] |
| FRPR-4 | FLP-13 | 67-541 nM | [[37](#_ENREF_37)] |
| **Somatostatin / Urotensin II receptor group** | | | |
| NPR-17 | FLP-24 | 0.1-1 μM | [[60](#_ENREF_60)] |
| **Gastrin / CCK-like receptor group** | | | |
| CKR-2 | NLP-12 | 15-30 nM | [[61](#_ENREF_61)] |
| **Vasopressin-like receptor group** | | | |
| NTR-1 | NTC-1 | 19 nM | [[43](#_ENREF_43)] |
| **Neurotensin / TPH-like receptor group** | | | |
| EGL-6 | FLP-10 | 11 nM | [[53](#_ENREF_53)] |
|  | FLP-17 | 1-28nM | [[53](#_ENREF_53)] |
| **Class B / Secretin receptor group** | | | |
| PDFR-1 | PDF-1 | 0.4-5 μM | [[41](#_ENREF_41)] |
|  | PDF-2 /  NLP-37 | 114 nM | [[41](#_ENREF_41)] |
