# Supplementary material for: The Multilayer Connectome of Caenorhabditis elegans
Source: PLoS Comput Biol. 2016 Dec 16;12(12):e1005283. doi: 10.1371/journal.pcbi.1005283 (PMC5215746; doi:10.1371/journal.pcbi.1005283)
Supplement: S11 Table — List of neurons connected by motif 9 (i.e. unidirectional OA link and synapse in reverse direction) or motif 11 (shaded, unidirectional OA link coincident with gap junction) (DOCX) [file pcbi.1005283.s015.docx]

| **Cell A** |  | **Cell B** |
| --- | --- | --- |
| RICL | ⟷ | ADLL |
| RICR | ⟷ | ADLR |
| RICL | ⟷ | CEP (DL/DR/VL/VR) |
| RICR | ⟷ | CEP (DL/DR/VL/VR) |
| RICL | ⟷ | ASHL |
| RICR | ⟷ | ASHR |
| RICL | ⟷ | AWBR |
