# Supplementary material for: The Multilayer Connectome of Caenorhabditis elegans
Source: PLoS Comput Biol. 2016 Dec 16;12(12):e1005283. doi: 10.1371/journal.pcbi.1005283 (PMC5215746; doi:10.1371/journal.pcbi.1005283)
Supplement: S12 Table — (DOCX) [file pcbi.1005283.s016.docx]

| Neuron | *k*_norm_ | *k*_syn_ | *k*_gap_ | *k*_ma_ | *k_np_* |
| --- | --- | --- | --- | --- | --- |
| RIMR⋆ | 0.164 | 34 | 14 | 128 | 114 |
| RIML⋆ | 0.120 | 28 | 12 | 128 | 114 |
| PVQR | 0.047 | 22 | 10 | 16 | 110 |
| ASHR | 0.046 | 21 | 12 | 10 | 162 |
| DVA⋆ | 0.046 | 54 | 10 | 8 | 104 |
| RIS | 0.036 | 27 | 16 | 14 | 44 |
| VD01 | 0.033 | 14 | 16 | 16 | 61 |
| ASHL | 0.033 | 18 | 10 | 10 | 162 |
| ADFR | 0.030 | 21 | 4 | 82 | 162 |
