# Supplementary material for: The Multilayer Connectome of Caenorhabditis elegans
Source: PLoS Comput Biol. 2016 Dec 16;12(12):e1005283. doi: 10.1371/journal.pcbi.1005283 (PMC5215746; doi:10.1371/journal.pcbi.1005283)
Supplement: S13 Table — Motif IDs correspond to those depicted in Figs 7 & S3. (DOCX) [file pcbi.1005283.s017.docx]

| **Motif ID** | **Frequency** | **Expected** | **Z-score** |
| --- | --- | --- | --- |
| 1 | 37076 | 36699 | 19.84 |
| 2 | 1358 | 1765 | -24.34 |
| 3 | 150 | 43 | 17.62 |
| 4 | 298 | 430 | -15.15 |
| 5 | 140 | 43 | 16.18 |
| 6 | 40 | 3 | 21.67 |
| 7 | 5404 | 5498 | -3.27 |
| 8 | 117 | 153 | -3.22 |
| 9 | 122 | 158 | -3.46 |
| 10 | 40 | 10 | 10.02 |
| 11 | 45 | 67 | -3.52 |
| 12 | 4 | 5 | -0.35 |
| 13 | 10 | 7 | 1.49 |
| 14 | 1 | 1 | -0.15 |
| 15 | 570 | 525 | 3.25 |
| 16 | 48 | 35 | 2.71 |
| 17 | 3 | 1 | 1.78 |
| 18 | 16 | 5 | 4.78 |
| 19 | 1 | 1 | -0.20 |
| 20 | 8 | 0 | 22.54 |
