# Supplementary material for: The Multilayer Connectome of Caenorhabditis elegans
Source: PLoS Comput Biol. 2016 Dec 16;12(12):e1005283. doi: 10.1371/journal.pcbi.1005283 (PMC5215746; doi:10.1371/journal.pcbi.1005283)
Supplement: S14 Table — List of neurons connected by motif 20 (i.e. reciprocal NP link, gap junction, and reciprocal synapses) (DOCX) [file pcbi.1005283.s018.docx]

| **Cell A** |  | **Cell B** |
| --- | --- | --- |
| FLPL | ⟷ | FLPR |
| PHAL | ⟷ | PHAR |
| PHBL | ⟷ | PHBR |
| RMGL | ⟷ | URXL |
| RMGR | ⟷ | URXR |
| RMGR | ⟷ | ASHR |
| PVR | ⟷ | DVA |
| AVAL | ⟷ | AVAR |
