# Supplementary material for: The Multilayer Connectome of Caenorhabditis elegans
Source: PLoS Comput Biol. 2016 Dec 16;12(12):e1005283. doi: 10.1371/journal.pcbi.1005283 (PMC5215746; doi:10.1371/journal.pcbi.1005283)
Supplement: S1 References — (DOCX) [file pcbi.1005283.s019.docx]

**Supplemental References**

1. Sze JY, Victor M, Loer C, Shi Y, Ruvkun G. Food and metabolic signaling defects in a *Caenorhabditis elegans* serotonin-synthesis mutant. Nature. 2000;403:560-4.

2. Pocock R, Hobert O. Hypoxia activates a latent circuit for processing gustatory information in C. elegans. Nature neuroscience. 2010;13(5):610-4. Epub 2010/04/20. doi: 10.1038/nn.2537. PubMed PMID: 20400959; PubMed Central PMCID: PMC3733994.

3. Jafari G, Xie Y, Kullyev A, Liang B, Sze JY. Regulation of extrasynaptic 5-HT by serotonin reuptake transporter function in 5-HT-absorbing neurons underscores adaptation behavior in Caenorhabditis elegans. The Journal of neuroscience : the official journal of the Society for Neuroscience. 2011;31(24):8948-57. Epub 2011/06/17. doi: 10.1523/JNEUROSCI.1692-11.2011. PubMed PMID: 21677178; PubMed Central PMCID: PMC3153855.

4. Suo S, Sasagawa N, Ishiura S. Cloning and characterization of a Caenorhabditis elegans D2-like dopamine receptor. Journal of neurochemistry. 2003;86(4):869-78. PubMed PMID: 12887685.

5. McDonald PW, Hardie SL, Jessen TN, Carvelli L, Matthies DS, Blakely RD. Vigorous motor activity in Caenorhabditis elegans requires efficient clearance of dopamine mediated by synaptic localization of the dopamine transporter DAT-1. The Journal of neuroscience : the official journal of the Society for Neuroscience. 2007;27(51):14216-27. Epub 2007/12/21. doi: 10.1523/JNEUROSCI.2992-07.2007. PubMed PMID: 18094261.

6. Alkema MJ, Hunter-Ensor M, Ringstad N, Horvitz HR. Tyramine Functions independently of octopamine in the Caenorhabditis elegans nervous system. Neuron. 2005;46(2):247-60. Epub 2005/04/26. doi: 10.1016/j.neuron.2005.02.024. PubMed PMID: 15848803.

7. Dernovici S, Starc T, Dent JA, Ribeiro P. The serotonin receptor SER-1 (5HT2ce) contributes to the regulation of locomotion in Caenorhabditis elegans. Dev Neurobiol. 2007;67(2):189-204. Epub 2007/04/20. doi: 10.1002/dneu.20340. PubMed PMID: 17443782.

8. Carnell L, Illi J, Hong SW, McIntire SL. The G-protein-coupled serotonin receptor SER-1 regulates egg laying and male mating behaviors in Caenorhabditis elegans. The Journal of neuroscience : the official journal of the Society for Neuroscience. 2005;25(46):10671-81. Epub 2005/11/18. doi: 10.1523/JNEUROSCI.3399-05.2005. PubMed PMID: 16291940.

9. Xiao H, Hapiak VM, Smith KA, Lin L, Hobson RJ, Plenefisch J, et al. SER-1, a Caenorhabditis elegans 5-HT2-like receptor, and a multi-PDZ domain containing protein (MPZ-1) interact in vulval muscle to facilitate serotonin-stimulated egg-laying. Developmental biology. 2006;298(2):379-91. Epub 2006/08/08. doi: 10.1016/j.ydbio.2006.06.044. PubMed PMID: 16890216.

10. Tsalik EL, Niacaris T, Wenick AS, Pau K, Avery L, Hobert O. LIM homeobox gene-dependent expression of biogenic amine receptors in restricted regions of the C. elegans nervous system. Developmental biology. 2003;263(1):81-102. Epub 2003/10/22. PubMed PMID: 14568548; PubMed Central PMCID: PMC4445141.

11. Gurel G, Gustafson MA, Pepper JS, Horvitz HR, Koelle MR. Receptors and other signaling proteins required for serotonin control of locomotion in Caenorhabditis elegans. Genetics. 2012;192(4):1359-71. Epub 2012/10/02. doi: 10.1534/genetics.112.142125. PubMed PMID: 23023001; PubMed Central PMCID: PMC3512144.

12. Shyn SI. Cameleon Reveals a Physiologic Correlate for Alternative Behavorial States in *C. Elegans* Egg-Laying: University of California, San Diego; 2003.

13. Hapiak VM, Hobson RJ, Hughes L, Smith K, Harris G, Condon C, et al. Dual excitatory and inhibitory serotonergic inputs modulate egg laying in Caenorhabditis elegans. Genetics. 2009;181(1):153-63. Epub 2008/11/13. doi: 10.1534/genetics.108.096891. PubMed PMID: 19001289; PubMed Central PMCID: PMC2621164.

14. Cunningham KA, Hua Z, Srinivasan S, Liu J, Lee BH, Edwards RH, et al. AMP-activated kinase links serotonergic signaling to glutamate release for regulation of feeding behavior in C. elegans. Cell Metab. 2012;16(1):113-21. Epub 2012/07/10. doi: 10.1016/j.cmet.2012.05.014. PubMed PMID: 22768843; PubMed Central PMCID: PMC3413480.

15. Hobson RJ, Hapiak VM, Xiao H, Buehrer KL, Komuniecki PR, Komuniecki RW. SER-7, a Caenorhabditis elegans 5-HT7-like receptor, is essential for the 5-HT stimulation of pharyngeal pumping and egg laying. Genetics. 2006;172(1):159-69. Epub 2005/10/06. doi: 10.1534/genetics.105.044495. PubMed PMID: 16204223; PubMed Central PMCID: PMC1456143.

16. Li Z, Li Y, Yi Y, Huang W, Yang S, Niu W, et al. Dissecting a central flip-flop circuit that integrates contradictory sensory cues in C. elegans feeding regulation. Nature communications. 2012;3:776. Epub 2012/04/12. doi: 10.1038/ncomms1780. PubMed PMID: 22491324.

17. Wragg RT, Hapiak V, Miller SB, Harris GP, Gray J, Komuniecki PR, et al. Tyramine and octopamine independently inhibit serotonin-stimulated aversive behaviors in Caenorhabditis elegans through two novel amine receptors. The Journal of neuroscience : the official journal of the Society for Neuroscience. 2007;27(49):13402-12. Epub 2007/12/07. doi: 10.1523/JNEUROSCI.3495-07.2007. PubMed PMID: 18057198.

18. Suo S, Kimura Y, Van Tol HH. Starvation induces cAMP response element-binding protein-dependent gene expression through octopamine-Gq signaling in Caenorhabditis elegans. The Journal of neuroscience : the official journal of the Society for Neuroscience. 2006;26(40):10082-90. PubMed PMID: 17021164.

19. Mills H, Hapiak V, Harris G, Summers P, Komuniecki R. The interaction of octopamine and neuropeptides to slow aversive responses in C. elegans mimics the modulation of chronic pain in mammals. Worm. 2012;1(4):202-6. Epub 2013/09/24. doi: 10.4161/worm.20467. PubMed PMID: 24058849; PubMed Central PMCID: PMC3670219.

20. Yoshida M, Oami E, Wang M, Ishiura S, Suo S. Nonredundant function of two highly homologous octopamine receptors in food-deprivation-mediated signaling in Caenorhabditis elegans. J Neurosci Res. 2014;92(5):671-8. Epub 2014/01/22. doi: 10.1002/jnr.23345. PubMed PMID: 24446241.

21. Sanyal S, Wintle RF, Kindt KS, Nuttley WM, Arvan R, Fitzmaurice P, et al. Dopamine modulates the plasticity of mechanosensory responses in Caenorhabditis elegans. The EMBO journal. 2004;23(2):473-82. Epub 2004/01/24. doi: 10.1038/sj.emboj.7600057. PubMed PMID: 14739932; PubMed Central PMCID: PMC1271763.

22. Chase DL, Pepper JS, Koelle MR. Mechanism of extrasynaptic dopamine signaling in Caenorhabditis elegans. Nature neuroscience. 2004;7(10):1096-103. Epub 2004/09/21. doi: 10.1038/nn1316. PubMed PMID: 15378064.

23. Etchberger JF, Flowers EB, Poole RJ, Bashllari E, Hobert O. Cis-regulatory mechanisms of left/right asymmetric neuron-subtype specification in C. elegans. Development. 2009;136(1):147-60. Epub 2008/12/09. doi: 10.1242/dev.030064. PubMed PMID: 19060335; PubMed Central PMCID: PMC2685964.

24. Suo S, Culotti JG, Van Tol HH. Dopamine counteracts octopamine signalling in a neural circuit mediating food response in C. elegans. The EMBO journal. 2009;28(16):2437-48. Epub 2009/07/18. doi: 10.1038/emboj.2009.194. PubMed PMID: 19609300; PubMed Central PMCID: PMC2735167.

25. Zhang F, Bhattacharya A, Nelson JC, Abe N, Gordon P, Lloret-Fernandez C, et al. The LIM and POU homeobox genes ttx-3 and unc-86 act as terminal selectors in distinct cholinergic and serotonergic neuron types. Development. 2014;141(2):422-35. Epub 2013/12/20. doi: 10.1242/dev.099721. PubMed PMID: 24353061; PubMed Central PMCID: PMC3879818.

26. Ezak MJ, Ferkey DM. The C. elegans D2-like dopamine receptor DOP-3 decreases behavioral sensitivity to the olfactory stimulus 1-octanol. PloS one. 2010;5(3):e9487. Epub 2010/03/09. doi: 10.1371/journal.pone.0009487. PubMed PMID: 20209143; PubMed Central PMCID: PMC2830454.

27. Sugiura M, Fuke S, Suo S, Sasagawa N, Van Tol HH, Ishiura S. Characterization of a novel D2-like dopamine receptor with a truncated splice variant and a D1-like dopamine receptor unique to invertebrates from Caenorhabditis elegans. Journal of neurochemistry. 2005;94(4):1146-57. PubMed PMID: 16001968.

28. Smith CJ, Watson JD, Spencer WC, O'Brien T, Cha B, Albeg A, et al. Time-lapse imaging and cell-specific expression profiling reveal dynamic branching and molecular determinants of a multi-dendritic nociceptor in C. elegans. Developmental biology. 2010;345(1):18-33. Epub 2010/06/12. doi: 10.1016/j.ydbio.2010.05.502. PubMed PMID: 20537990; PubMed Central PMCID: PMC2919608.

29. Rex E, Molitor SC, Hapiak V, Xiao H, Henderson M, Komuniecki R. Tyramine receptor (SER-2) isoforms are involved in the regulation of pharyngeal pumping and foraging behavior in Caenorhabditis elegans. Journal of neurochemistry. 2004;91(5):1104-15. Epub 2004/12/01. doi: 10.1111/j.1471-4159.2004.02787.x. PubMed PMID: 15569254.

30. Donnelly JL, Clark CM, Leifer AM, Pirri JK, Haburcak M, Francis MM, et al. Monoaminergic orchestration of motor programs in a complex C. elegans behavior. PLoS biology. 2013;11(4):e1001529. Epub 2013/04/09. doi: 10.1371/journal.pbio.1001529. PubMed PMID: 23565061; PubMed Central PMCID: PMC3614513.

31. Rex E, Hapiak V, Hobson R, Smith K, Xiao H, Komuniecki R. TYRA-2 (F01E11.5): a Caenorhabditis elegans tyramine receptor expressed in the MC and NSM pharyngeal neurons. Journal of neurochemistry. 2005;94(1):181-91. Epub 2005/06/15. doi: 10.1111/j.1471-4159.2005.03180.x. PubMed PMID: 15953361.

32. Bendesky A, Tsunozaki M, Rockman MV, Kruglyak L, Bargmann CI. Catecholamine receptor polymorphisms affect decision-making in C. elegans. Nature. 2011;472(7343):313-8. Epub 2011/03/18. doi: 10.1038/nature09821. PubMed PMID: 21412235; PubMed Central PMCID: PMC3154120.

33. Hunt-Newbury R, Viveiros R, Johnsen R, Mah A, Anastas D, Fang L, et al. High-throughput in vivo analysis of gene expression in Caenorhabditis elegans. PLoS biology. 2007;5(9):e237. Epub 2007/09/14. doi: 10.1371/journal.pbio.0050237. PubMed PMID: 17850180; PubMed Central PMCID: PMC1971126.

34. Pirri JK, McPherson AD, Donnelly JL, Francis MM, Alkema MJ. A tyramine-gated chloride channel coordinates distinct motor programs of a Caenorhabditis elegans escape response. Neuron. 2009;62(4):526-38. Epub 2009/05/30. doi: 10.1016/j.neuron.2009.04.013. PubMed PMID: 19477154; PubMed Central PMCID: PMC2804440.

35. Ringstad N, Abe N, Horvitz HR. Ligand-gated chloride channels are receptors for biogenic amines in C. elegans. Science. 2009;325(5936):96-100. Epub 2009/07/04. doi: 10.1126/science.1169243. PubMed PMID: 19574391; PubMed Central PMCID: PMC2963310.

36. Kim K, Li C. Expression and regulation of an FMRFamide-related neuropeptide gene family in Caenorhabditis elegans. J Comp Neurol. 2004;475(4):540-50. PubMed PMID: 15236235.

37. Nelson MD, Janssen T, York N, Lee KH, Schoofs L, Raizen DM. FRPR-4 Is a G-Protein Coupled Neuropeptide Receptor That Regulates Behavioral Quiescence and Posture in Caenorhabditis elegans. PloS one. 2015;10(11):e0142938. Epub 2015/11/17. doi: 10.1371/journal.pone.0142938. PubMed PMID: 26571132; PubMed Central PMCID: PMC4646455.

38. Macosko EZ, Pokala N, Feinberg EH, Chalasani SH, Butcher RA, Clardy J, et al. A hub-and-spoke circuit drives pheromone attraction and social behaviour in C. elegans. Nature. 2009;458(7242):1171-5. Epub 2009/04/08. doi: 10.1038/nature07886. PubMed PMID: 19349961; PubMed Central PMCID: PMC2760495.

39. Nathoo AN, Moeller RA, Westlund BA, Hart AC. Identification of neuropeptide-like protein gene families in Caenorhabditiselegans and other species. Proceedings of the National Academy of Sciences of the United States of America. 2001;98(24):14000-5. Epub 2001/11/22. doi: 10.1073/pnas.241231298. PubMed PMID: 11717458; PubMed Central PMCID: PMC61156.

40. Karakuzu O, Wang DP, Cameron S. MIG-32 and SPAT-3A are PRC1 homologs that control neuronal migration in Caenorhabditis elegans. Development. 2009;136(6):943-53. Epub 2009/02/13. doi: 10.1242/dev.029363. PubMed PMID: 19211678; PubMed Central PMCID: PMC2727560.

41. Janssen T, Husson SJ, Lindemans M, Mertens I, Rademakers S, Ver Donck K, et al. Functional characterization of three G protein-coupled receptors for pigment dispersing factors in Caenorhabditis elegans. The Journal of biological chemistry. 2008;283(22):15241-9. Epub 2008/04/09. doi: 10.1074/jbc.M709060200. PubMed PMID: 18390545; PubMed Central PMCID: PMC3258896.

42. Garrison JL, Macosko EZ, Bernstein S, Pokala N, Albrecht DR, Bargmann CI. Oxytocin/vasopressin-related peptides have an ancient role in reproductive behavior. Science. 2012;338(6106):540-3. Epub 2012/11/01. doi: 10.1126/science.1226201. PubMed PMID: 23112335; PubMed Central PMCID: PMC3597094.

43. Beets I, Janssen T, Meelkop E, Temmerman L, Suetens N, Rademakers S, et al. Vasopressin/oxytocin-related signaling regulates gustatory associative learning in C. elegans. Science. 2012;338(6106):543-5. Epub 2012/11/01. doi: 10.1126/science.1226860. PubMed PMID: 23112336.

44. Barrios A, Ghosh R, Fang C, Emmons SW, Barr MM. PDF-1 neuropeptide signaling modulates a neural circuit for mate-searching behavior in C. elegans. Nature neuroscience. 2012;15(12):1675-82. Epub 2012/11/13. doi: 10.1038/nn.3253. PubMed PMID: 23143519; PubMed Central PMCID: PMC3509246.

45. Coates JC, de Bono M. Antagonistic pathways in neurons exposed to body fluid regulate social feeding in Caenorhabditis elegans. Nature. 2002;419(6910):925-9. PubMed PMID: 12410311.

46. Luo J, Xu Z, Tan Z, Zhang Z, Ma L. Neuropeptide receptors NPR-1 and NPR-2 regulate Caenorhabditis elegans avoidance response to the plant stress hormone methyl salicylate. Genetics. 2015;199(2):523-31. Epub 2014/12/21. doi: 10.1534/genetics.114.172239. PubMed PMID: 25527285; PubMed Central PMCID: PMC4317659.

47. Keating CD, Kriek N, Daniels M, Ashcroft NR, Hopper NA, Siney EJ, et al. Whole-genome analysis of 60 G protein-coupled receptors in Caenorhabditis elegans by gene knockout with RNAi. Current biology : CB. 2003;13(19):1715-20. PubMed PMID: 14521838.

48. Cohen M, Reale V, Olofsson B, Knights A, Evans P, de Bono M. Coordinated regulation of foraging and metabolism in C. elegans by RFamide neuropeptide signaling. Cell Metab. 2009;9(4):375-85. Epub 2009/04/10. doi: 10.1016/j.cmet.2009.02.003. PubMed PMID: 19356718.

49. Chalasani SH, Kato S, Albrecht DR, Nakagawa T, Abbott LF, Bargmann CI. Neuropeptide feedback modifies odor-evoked dynamics in Caenorhabditis elegans olfactory neurons. Nature neuroscience. 2010;13(5):615-21. Epub 2010/04/07. doi: 10.1038/nn.2526. PubMed PMID: 20364145; PubMed Central PMCID: PMC2937567.

50. Harris G, Mills H, Wragg R, Hapiak V, Castelletto M, Korchnak A, et al. The monoaminergic modulation of sensory-mediated aversive responses in Caenorhabditis elegans requires glutamatergic/peptidergic cotransmission. The Journal of neuroscience : the official journal of the Society for Neuroscience. 2010;30(23):7889-99. Epub 2010/06/11. doi: 10.1523/JNEUROSCI.0497-10.2010. PubMed PMID: 20534837; PubMed Central PMCID: PMC3005568.

51. Wenick AS, Hobert O. Genomic cis-regulatory architecture and trans-acting regulators of a single interneuron-specific gene battery in C. elegans. Developmental cell. 2004;6(6):757-70. Epub 2004/06/05. doi: 10.1016/j.devcel.2004.05.004. PubMed PMID: 15177025.

52. Hu Z, Pym EC, Babu K, Vashlishan Murray AB, Kaplan JM. A neuropeptide-mediated stretch response links muscle contraction to changes in neurotransmitter release. Neuron. 2011;71(1):92-102. Epub 2011/07/13. doi: 10.1016/j.neuron.2011.04.021. PubMed PMID: 21745640; PubMed Central PMCID: PMC3134788.

53. Ringstad N, Horvitz HR. FMRFamide neuropeptides and acetylcholine synergistically inhibit egg-laying by C. elegans. Nature neuroscience. 2008;11(10):1168-76. Epub 2008/09/23. doi: 10.1038/nn.2186. PubMed PMID: 18806786; PubMed Central PMCID: PMC2963311.

54. Rogers C, Reale V, Kim K, Chatwin H, Li C, Evans P, et al. Inhibition of Caenorhabditis elegans social feeding by FMRFamide-related peptide activation of NPR-1. Nature neuroscience. 2003;6(11):1178-85. PubMed PMID: 14555955.

55. Kubiak TM, Larsen MJ, Nulf SC, Zantello MR, Burton KJ, Bowman JW, et al. Differential activation of "social" and "solitary" variants of the Caenorhabditis elegans G protein-coupled receptor NPR-1 by its cognate ligand AF9. The Journal of biological chemistry. 2003;278(36):33724-9. Epub 2003/06/25. doi: 10.1074/jbc.M304861200. PubMed PMID: 12821653.

56. Ezcurra M, Walker DS, Beets I, Swoboda P, Schafer WR. Neuropeptidergic Signaling and Active Feeding State Inhibit Nociception in Caenorhabditis elegans. The Journal of neuroscience : the official journal of the Society for Neuroscience. 2016;36(11):3157-69. Epub 2016/03/18. doi: 10.1523/JNEUROSCI.1128-15.2016. PubMed PMID: 26985027; PubMed Central PMCID: PMC4792932.

57. Kubiak TM, Larsen MJ, Zantello MR, Bowman JW, Nulf SC, Lowery DE. Functional annotation of the putative orphan Caenorhabditis elegans G-protein-coupled receptor C10C6.2 as a FLP15 peptide receptor. The Journal of biological chemistry. 2003;278(43):42115-20. Epub 2003/08/26. doi: 10.1074/jbc.M304056200. PubMed PMID: 12937167.

58. Geary T, Kubiak T, Larsen M, Lowery D, inventorsG protein-coupled receptor like receptors and modulators thereof2002.

59. Kubiak TM, Larsen MJ, Bowman JW, Geary TG, Lowery DE. FMRFamide-like peptides encoded on the flp-18 precursor gene activate two isoforms of the orphan Caenorhabditis elegans G-protein-coupled receptor Y58G8A.4 heterologously expressed in mammalian cells. Biopolymers. 2008;90(3):339-48. Epub 2007/09/20. doi: 10.1002/bip.20850. PubMed PMID: 17879267.

60. Cheong MC, Artyukhin AB, You YJ, Avery L. An opioid-like system regulating feeding behavior in C. elegans. Elife. 2015;4. Epub 2015/04/22. doi: 10.7554/eLife.06683. PubMed PMID: 25898004; PubMed Central PMCID: PMC4427864.

61. Janssen T, Meelkop E, Lindemans M, Verstraelen K, Husson SJ, Temmerman L, et al. Discovery of a cholecystokinin-gastrin-like signaling system in nematodes. Endocrinology. 2008;149(6):2826-39. Epub 2008/03/15. doi: 10.1210/en.2007-1772. PubMed PMID: 18339709.
